# pH-Responsive Triplex DNA Nanoswitches: Surface Plasmon Resonance Platform for Bladder Cancer-Associated microRNAs

**DOI:** 10.1021/acsnano.4c16396

**Published:** 2025-02-12

**Authors:** Pei-Ying Lin, Ying-Feng Chang, Cheng-Che Chen, Li-Chen Su, Itamar Willner, Ja-an Annie Ho

**Affiliations:** † Department of Biochemical Science and Technology, National Taiwan University, Taipei 10617, Taiwan; ‡ Artificial Intelligence Research Center, Chang Gung University, Taoyuan 33302, Taiwan; § Department of Gastroenterology and Hepatology, New Taipei Municipal Tucheng Hospital (Built and Operated by Chang Gung Medical Foundation), New Taipei City 23652, Taiwan; ∥ Department of Urology, Taichung Veterans General Hospital, Taichung 40705, Taiwan; ⊥ Department of Medicine and Nursing, HungKuang University, Taichung 43304, Taiwan; # Organic Electronics Research Center, Ming Chi University of Technology, New Taipei City 243303, Taiwan; ∇ General Education Center, Ming Chi University of Technology, New Taipei City 243303, Taiwan; ○ Institute of Chemistry, The Hebrew University of Jerusalem, Jerusalem 91904, Israel; ◆ Department of Chemistry, National Taiwan University, Taipei 10617, Taiwan; ¶ Center for Emerging Materials and Advanced Devices, National Taiwan University, Taipei 10617, Taiwan; ▶ Center for Biotechnology, National Taiwan University, Taipei 10617, Taiwan

**Keywords:** biosensor, antibody, multiplex analysis, Au nanoparticle, DNA nanoswitch

## Abstract

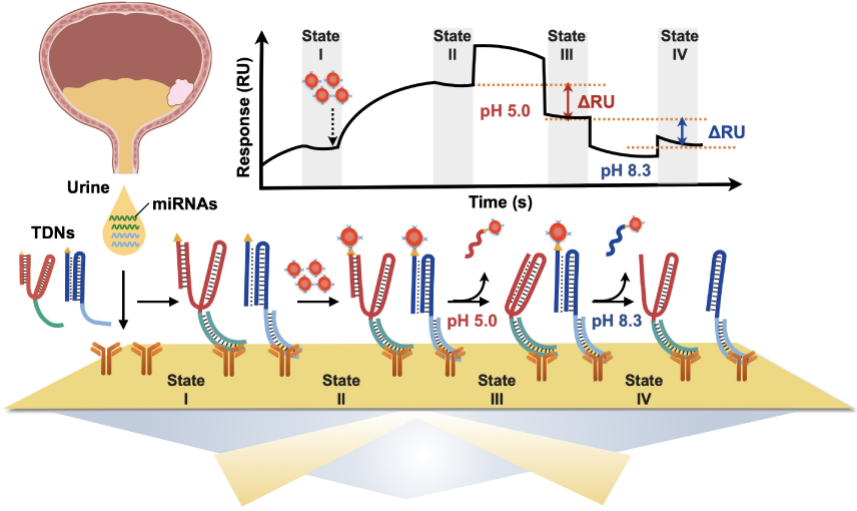

Bladder cancer (BC) has a high recurrence rate, necessitating frequent monitoring. We herein present an innovative method for detecting BC-related miR-183 and miR-155 microRNAs using pH-responsive triplex DNA nanoswitches (TDNs). This approach employs a stepwise surface plasmon resonance biosensing platform (TDNs-SPR assay) to detect these two miRNAs sequentially. The platform involves the assembly of two triplex pH-responsive probes, switch A (SA) and switch B (SB), on an SPR sensing interface by anchoring the probes to the surface through SA/miR-183 and SB/miR-155 binding to the S9.6 antibody-modified surface. The probes are functionalized with streptavidin-Au nanoparticles/biotinylated strands, which act as reporter units for the presence of the respective miRNAs on the sensing interface. The pH-induced displacement of reporter units triggers stepwise SPR reflectivity changes: at pH 5.0 for sensing miR-183 and at pH 8.3 for sensing miR-155. The reflectivity changes relate quantitatively to the concentrations of miRNAs. This sensing platform enables the detection of two miRNAs with detection limits as low as 0.57 pM for miR-183 and 0.83 pM for miR-155, highlighting its powerful utility for precise biomarker analysis. Moreover, this platform distinguishes BC patients from healthy individuals in urine samples. The method offers a versatile, noninvasive method for detecting any two miRNAs associated with other diseases.

## Introduction

Triplex DNAs, formed by Hoogsteen base pairing, involve the insertion of a third strand into specific double-strand DNA.^1^ It has been reported that C-G·C^+^ triplets require the protonation of cytosine in the third strand under acidic pH conditions, while T-A·T triplets remain stable across acidic to neutral pH and destabilized at basic pH, due to the deprotonation of thymine.^2^ The pH responsiveness of triplex DNA nanoswitches (TDNs), controlled by regulating the T-A·T and C-G·C^+^ ratios in the sequence, enables the construction of programmable DNA nanoswitches.^3^ Diverse applications of reconfiguration of triplex structures were reported, including pH-controlled drug release,^4,5^ pH-responsive hydrogel,^6^ and pH-induced nanoparticle aggregation.^7^ Moreover, triplex structures have been utilized in assembling biosensing platforms^8^ for detecting protein activity,^9^ small molecules,^10^ and nucleic acid.^11,12^ However, their application in multiplex nucleic acids sensing platforms has yet to be explored.

Bladder cancer (BC) ranks as the tenth most prevalent malignancy globally, with an incidence of 610,000 new cases reported in 2020 and an anticipated 65% increase by 2045, as projected by the World Health Organization.^13,14^ The necessity for early detection is paramount, given BC’s high recurrence rate, which significantly influences the effectiveness of treatment and monitoring strategies.^15^ Cystoscopy, though the gold standard for BC diagnosis, is an invasive procedure and carries the potential to overlook carcinoma in situ.^16^ Conversely, urine cytology, a noninvasive diagnostic method, is hindered by its low sensitivity.^17^ Consequently, there is a critical need for the development of noninvasive and highly specific biosensors to improve the accuracy and reliability of BC diagnosis.^18^ MicroRNAs (miRNAs), small noncoding RNAs involved in post-transcriptional gene regulation,^19,20^ are promising biomarkers for BC.^21,22^ Yamada et al. reported an upregulation of miR-183–5p in the urine of BC patients compared to healthy controls, correlating with tumor stage.^23−25^ Similarly, miR-155 is overexpressed in BC patients compared to both healthy controls and patients with bladder inflammation and is associated with higher recurrence rates.^26^ Studies also indicated that utilizing multiplex urinary BC-associated miRNAs could enhance diagnostic accuracy.^27,28^ Accordingly, urinary miR-183 and miR-155 emerged as potential diagnostic and prognosis biomarkers for BC.

Quantifying miRNAs presents significant challenges due to their short length, high sequence homology, and low abundance in body fluids.^29^ Moreover, because a single miRNA can regulate multiple genes, relying on a single miRNA as a biomarker may be insufficient for reliable disease detection.^30^ Thus, a multi-miRNA analysis is necessary to ensure accurate and reliable diagnosis. Currently, quantitative real-time polymerase chain reaction (qRT-PCR) serves as the gold standard for miRNA detection due to its high sensitivity. However, this method is constrained by the need for repetitive thermal cycling, a labor-intensive protocol, and limited capacity for multiplexing.^31^ On the other hand, microarrays provide high-throughput screening for multiple miRNAs but display relatively low sensitivity.^32^ Therefore, there is a growing interest in developing isothermal and multiplex miRNA biosensors.^33^ Various approaches, including different fluorophores,^34,35^ Quantum dots,^36^ Raman spectroscopy,^37^ electrochemical redox tags,^38^ electrochemiluminescence luminophores,^39,40^ metal nanoparticles,^41^ and functional DNA nanostructures^42^ have been employed as multiple signal readouts for miRNA sensing. However, approaches with multilabels often suffer from signal overlap and limited label availability.

Surface plasmon resonance (SPR) biosensors offer rapid, label-free, highly sensitive analysis of biomolecule interaction in real time.^43,44^ Various SPR-based miRNA biosensors have been reported.^44,45^ However, due to the low molecular weight of miRNAs, signal amplification strategies are required to enhance the mass change on the sensing chip and induce the variation in refractive index.^45^ These strategies include DNA supersandwich assembly,^46^ catalytic hairpin assembly,^47^ enzyme amplification reaction,^48^ and nanoparticles enhancement.^49,50^ Moreover, the labeling of SPR sensing events with plasmonic nanoparticles resulted in coupling between the plasmonic nanoparticles and the surface plasmon wave, resulting in enhanced SPR shifts and superior amplified analytical sensitivities.^51^ However, some of these approaches are complex, time-consuming, and involve intricate probe design and enzyme participation. Additionally, distinguishing SPR responses from different analytes within a single sensing channel is challenging, since the SPR response is related to the refractive-index changes on the surface. To enable multiplexing in SPR biosensors, sensor chips with multichannels or SPR imaging systems are generally employed.^52,53^ However, this approach requires additional ligand modification for each channel, which potentially increases costs, and lengthens sensing times. Motivated by the versatile and pH-responsive triplex DNA nanostructure, we integrated it with AuNPs to create a switchable sensing probe for developing an SPR assay capable of multiplex miRNA detection.

Herein, we report the development of a nucleic-acid amplification-free SPR biosensor using triplex DNA nanoswitches (TDNs-SPR assay) for the dual-detection of BC-associated miRNAs. Two TDNs with distinct pH responsiveness were designed to target two miRNAs (miR-183 and miR-155). The TDNs/miRNA mixtures were introduced into an SPR chip immobilized with an antibody that recognizes DNA/RNA hybrids, followed by the binding of AuNPs to the TDNs/miRNA complex, thereby enhancing the SPR transduction signal. Multiplex signal acquisition was achieved by adjusting the pH of reaction buffers to induce the transformation of TDNs, leading to the detachment of AuNP-labeled reporter units from the sensing surface. The release of AuNP-labeled reporter units caused changes in SPR response at corresponding pH values, which were proportional to the miRNA concentrations. Furthermore, we introduce a method to minimize nonspecific perturbing adsorption effects. The platform was validated through the analysis of urine samples from BC patients and healthy controls, with results compared to those obtained using a commercial qRT-PCR assay.

## Results and Discussion

### Principle of TDNs-SPR Platform for Dual-Detection of microRNAs

**Scheme 1:**
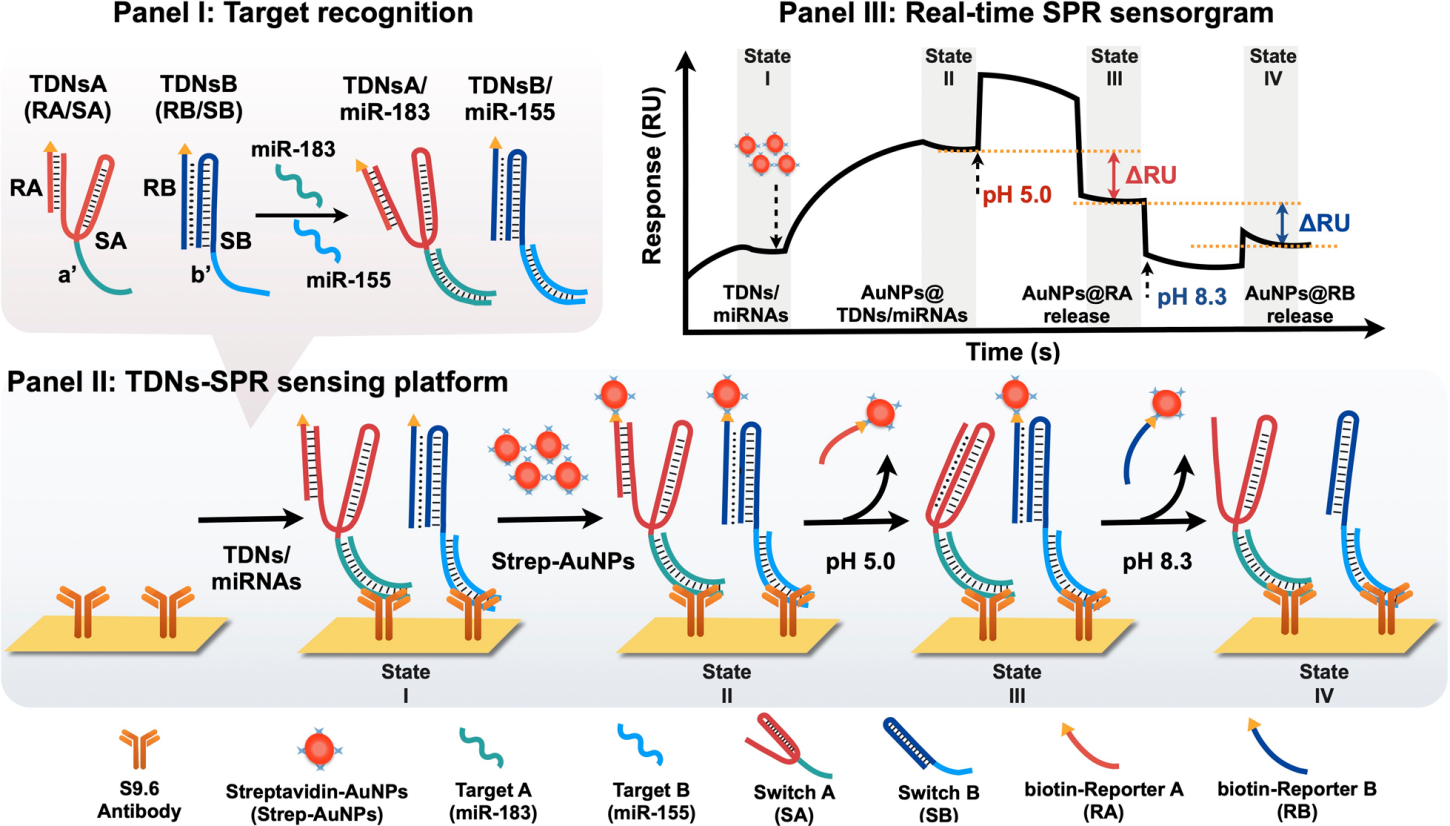
Schematic Illustration of pH-Responsive Triplex DNA Nanoswitches (TDNs) in Manipulating the Release of AuNPs-Labeled Reporter Unit on SPR Platform for Dual-Detection of Bladder Cancer-Associated miRNA Target miRNA recognized by TDNs, resulting in the formation of TDNs/miRNA complexes, is illustrated in Panel I. These TDNs/miRNAs complexes were subsequently introduced into the SPR sensing system, followed by sequential injections of AuNPs and buffers of pH 5.0 and pH 8.3, respectively, as shown in Panel II. Panel III presents the SPR real-time sensorgram, depicting the response curve for the sensing process.

The TDNs-SPR sensing platform and its operation are schematically presented in Scheme 1. It includes two pH-responsive switchable sensing modules, switch A (SA) and switch B (SB). As depicted in **Panel I** of Scheme 1, while module SA is blocked by the biotinylated reporter unit A (RA), forming the TDNsA (RA/SA) complex, module SB is engineered to consist of a triplex structure that includes the biotinylated reporter unit B (RB), resulting in the formation of TDNsB (RB/SB). The reporter-modified units are pH-responsive. The RA-capped SA can reconfigure at acidic pH to form a C-G·C^+^ triplex while releasing the RA unit. In contrast, the RB-functionalized SB module is a T-A·T structure that dissociates into a TA duplex under basic conditions, releasing the RB unit. The two sensing modules include single-stranded DNA tethers a’ and b’ complementary to the target miRNA analytes, miRNA-183 and miRNA-155, acting as biomarkers for BC. In the presence of the two biomarkers, duplexes miRNA-183/a’ and miRNA-155/b’ are formed. The operation of the pH-responsive TDNs-SPR sensing platform is schematically displayed in **Panel II**. The SPR surface is functionalized with S9.6 antibodies, specifically binding DNA/RNA duplexes. Subjecting the antibody-modified SPR surface to the mixture of TDNsA/miRNA-183 and TDNsB/miRNA-155 results in the active sensing interface for dual, triplex-based, pH-switchable detection of the two miRNAs (**State I**). Treatment of the sensing interface with streptavidin-conjugated Au nanoparticles (Strep-AuNPs) results in the binding of Strep-AuNPs to the biotin labels associated with RA and RB (**State II**). The AuNPs act as amplifying labels of the SPR signals as a result of the plasmonic coupling of the nanoparticles with the SPR wave associated with the surface. Treatment of the resulting sensing interface at pH 5.0 leads to a reconfiguration of SA into the C-G·C^+^ triplex structure, while releasing the AuNPs-labeled RA (AuNPs@RA). The release of the AuNPs@RA is then, accompanied by a time-dependent intensity decrease of the SPR reflectivity (**State III**). The extent of reflectivity change relates to the concentration/surface coverage of the SA/miRNA-183 on the surface. Similarly, subsequent switching the pH of the solution to pH 8.3, separated the AuNPs-labeled RB (AuNPs@RB) associated with the sensing module SB, resulting in a further time-dependent decrease in intensity of the SPR reflectivity signal (**State IV**). Again, the extent of intensity decrease of the SPR reflectivity signal relates to the concentration/surface coverage of the SB/miRNA-155 associated with the surface. **Panel III** schematically illustrates the changes in SPR reflectivity intensity during the operation of the sensing platform. The sequences comprising the pH-responsive triplex nanoswitches are listed in Table 1. A detailed description of the assembly of the sensing platform, and the analytical procedures applied to characterize the system are provided in the material and methods.

**Table 1:** Sequences of the pH-Responsive Triplex DNA Nanoswitches^a^

Name	Sequence (5′→3′)
Target A (miR-183)	UAUGGCACUGGUAGAAUUCACU
Target B (miR-155)	UUAAUGCUAAUCGUGAUAGGGGU
Reporter A	Biotin-TTTACGGAGGGGAGG
Reporter B (70% TAT)	TTCTTTTCTCTTCTTCTCTTTTT-Biotin
Switch A (30% TAT)	**AGTGAATTCTACCAGTGCCATA**GAGGAGAGGAGAGGAGGGGAGGTTTACCTCCCCTCCTCTCCTCTCCCTTTGCCTCTCCTCTCCTCCCCTCCGT
Switch B (70% TAT)	AAGAAAAGAGAAGAAGAGAACACCTTCTCTTCTTCTCTTTTCTTAG**ACCCCTATCACGATTAGCATTAA**

a The bold letters represent the complementary sequences to the corresponding miRNAs. The underlined bases represent the triplex sequences.

The novelty of the analytical platform is reflected by several elements: (i) A method for capturing dual miRNAs on the SPR surface using anchored antibodies specific to DNA/miRNA duplexes. (ii) The stepwise detection of two bladder cancer-related miRNAs is achieved via the coassembly of two pH-responsive C-G·C^+^ and T-A·T triplex DNA nanoswitches on an SPR sensing interface, providing selective and reliable detection of the target miRNAs. (iii) Enhancement of analytical sensitivities is achieved through coupling between plasmonic particles and the surface plasmon wave. (iv) The versatility of the sensing platform allows the adaptation to any other target miRNAs.

### Evaluation of the Binding Features between the S9.6 Antibody and the pH-Switchable DNA/miRNA Hybrids

**Figure 1:**
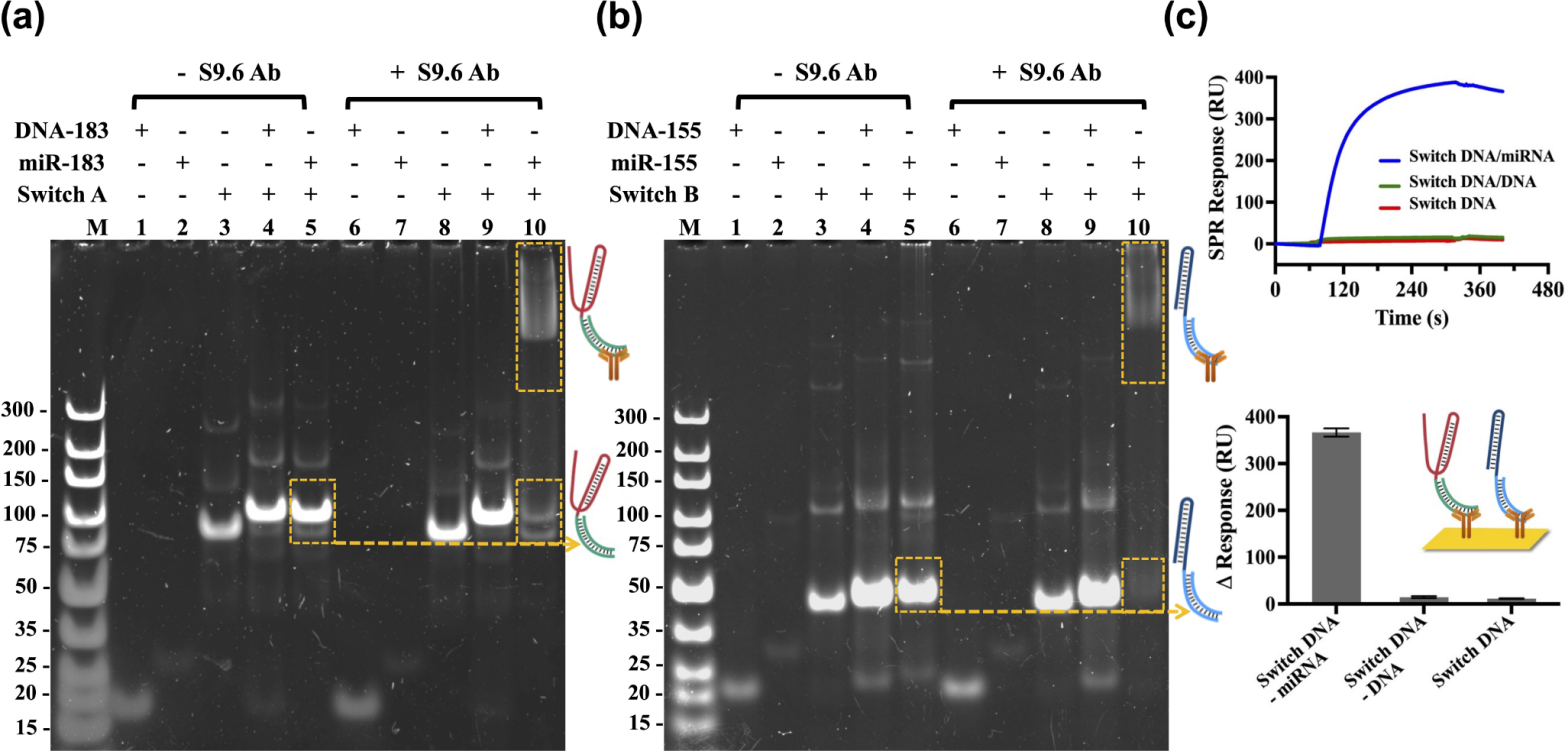
Evaluation of the binding between S9.6 antibody and DNA/miRNA hybrid switches. (a) Gel electrophoretic analysis of the binding between S9.6 antibody and switch A/miR-183 hybrid. Lane M: DNA marker (15 to 300 bp). Lanes 1 to 5: target DNA, target RNA, switch DNA, switch DNA/target DNA hybrids, and switch DNA/target miRNA hybrids, respectively. Lanes 6 to 10: same nucleic acids as lanes 1 to 5, incubated with S9.6 antibody at room temperature for 30 min. (b) Gel electrophoretic analysis of the binding between S9.6 antibody and switch B/miR-155 hybrid. Lane assignments are the same as in (a). (c) SPR analysis of the binding between S9.6 antibody and switch DNA/target miRNA hybrids (blue), switch DNA/target DNA hybrids (green), and only switch DNA (red). Samples were prepared by mixing switch A/miR-183 (or DNA-183) with switch B/miR-155 (or DNA-155) hybrids. All nucleic acids used in gel electrophoresis were 200 nM, and the S9.6 antibody was used at 80 μg/mL.

The S9.6 antibody, known for its specific binding to DNA/RNA hybrids,^54−57^ was selected as the biorecognition element and it was assembled on an SPR sensing chip to capture the TDNs/miRNA complex. Its nucleotide sequence-independent binding properties make it ideal for multiplex miRNA detection.^36,58^ In addition, Liu et al.^59^ demonstrated the stability of S9.6 antibody/DNA/miRNA complexes under liquid flow conditions, with binding occurring within minutes, offering a faster alternative to conventional nucleic acid hybridization methods. The binding specificity of the S9.6 antibody toward nucleic acids was initially evaluated through electrophoretic mobility shift assay (EMSA). In Figure 1a,b, lanes 1 to 5 show nucleic acids alone, while lanes 6 to 10 present nucleic acids incubated with the S9.6 antibody. No interaction with the S9.6 antibody was detected in lanes 6 to 9, which contain either single- or double-stranded DNA. However, a distinct band shift was observed in lane 10, where the DNA/miRNA hybrid switch was present, confirming the specific binding affinity of the S9.6 antibody to these hybrids.

Subsequently, the S9.6 antibody was immobilized on the SPR CM5 sensing chip using the commercial amine coupling reaction (see material and methods for details). As shown in Figure 1c, successful binding of the S9.6 antibodies to the pH-switchable DNA/miRNA hybrids was observed, evidenced by the temporal changes in SPR reflectivity intensity. In contrast, no interactions were detected between the antibody and either the analogous DNA/DNA duplex or the single tether TDNs module. These results are consistent with previous reports.^55,60^ Additionally, a single-cycle kinetics assay revealed that the S9.6 antibody exhibited comparable binding kinetics toward the two DNA/miRNA hybrid switches (SA/miR-183, SB/miR-155) at pH 7.0 with the K_D_ values corresponding to 2.91 × 10^–10^ M and 1.44 × 10^–10^ M, respectively (Table S2). These findings confirm the antibody’s high-affinity recognition capability for multiple DNA/miRNA hybrid switches.

Given the crucial role of pH in controlling the transition of TDNs, we investigated the pH independence of the DNA/miRNA hybrid switches in their interaction with the S9.6 antibody. Initially, we employed a DNA-based target that mimics the RNA target, labeling it with the pH-insensitive fluorophore TAMRA (Figure S1).^61^ The switch DNA was also labeled with the quencher BHQ-2 at the end of the sequence. Results demonstrated that the switch DNA and target DNA could successfully form hybrids across a pH range of 5.0 to 9.0 (Figure S2). Due to the pH tolerance of S9.6 antibody (up to pH 8.5),^60^ we selected a pH range below this threshold for further experiments. In the SPR system, no significant changes in responses were observed when the pH 5.0 and pH 8.3 releasing buffers were introduced across the DNA/miRNA hybrid switches captured by the S9.6 antibodies on the chip. This observation indicates that the binding affinity of the DNA/miRNA hybrid switches was not influenced by these buffer conditions (Figure S3).^60^ Moreover, HEPES buffer containing 15 mM Mg^2+^ and 80 mM NaCl at pH 7.0 was identified as the optimal running buffer for subsequent SPR experiments (Figure S4). The presence of Mg^2+^ is likely to enhance the stability of DNA/RNA hybrids, thus improving the performance of the SPR assay.^54^

For the regeneration test, the S9.6 antibody-immobilized chip demonstrated reuse capability for a minimum of five cycles, following sequential injections of 10 mM Glycine-HCl (pH 1.7) and 2 M MgCl_2_ (Figure S5). The coefficient of variation (CV) for five repeated binding responses of DNA/miRNA hybrid switches was below 3.4%, indicating the high affinity and reusability of the S9.6 antibody as a ligand for miRNA sensing on the SPR platform.

### Optimization of pH-Responsive Triplex DNA Nanoswitches for miRNA Detection

**Figure 2:**
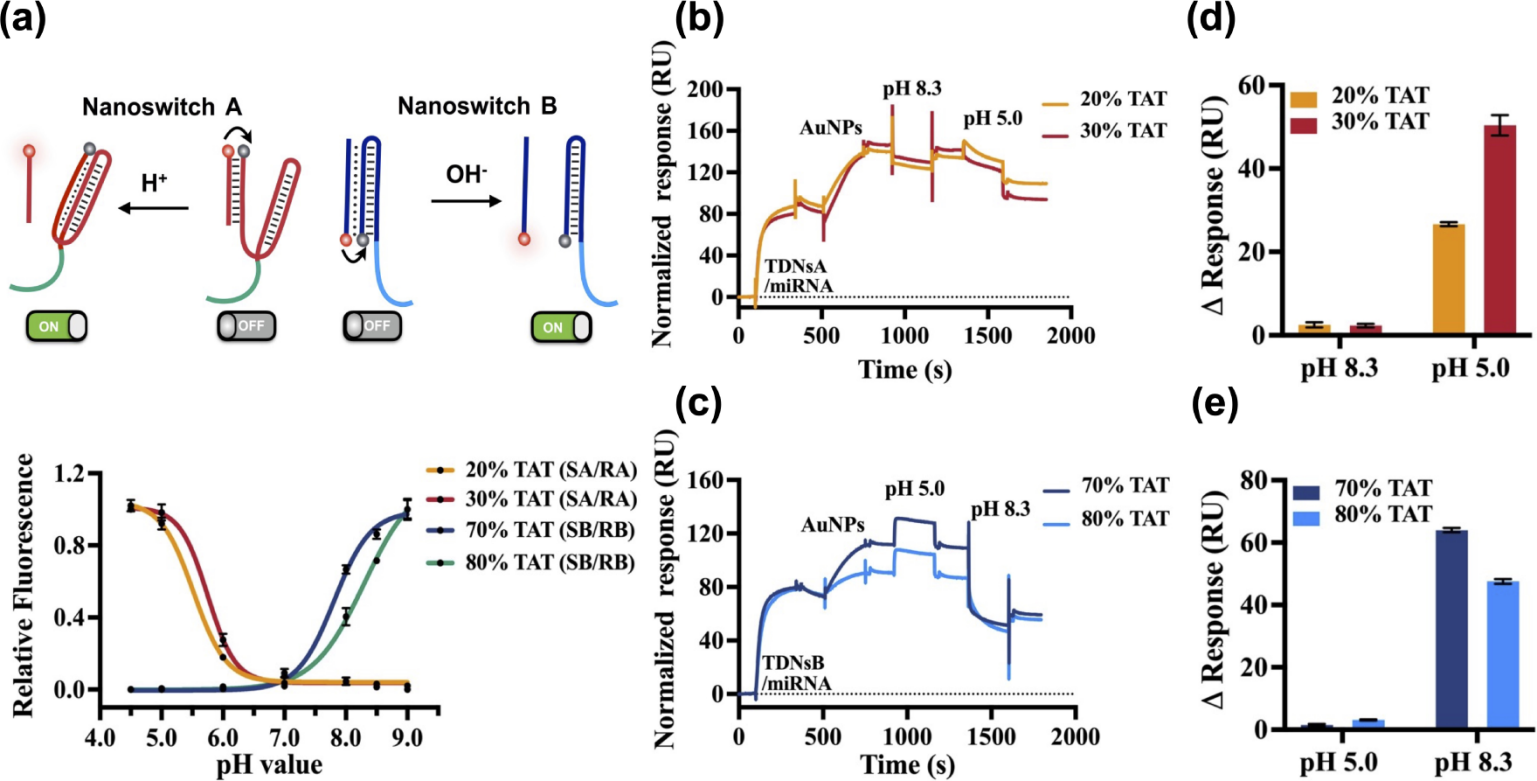
Optimization of pH-responsive triplex DNA nanoswitches for miRNA detection. (a) Fluorescence measurement of the pH-responsive performance of nanoswitches using fluorophore and quencher pairs. (b, c) pH-responsive performance of nanoswitches functionalized with AuNPs for target miRNA detection on the SPR platform. (d, e) The changes in SPR reflectivity (ΔRU) due to the release of AuNP-labeled reporter units at pH 5.0 and pH 8.3 for TDNsA and TDNsB, respectively. The ΔRU was determined by comparing the response unit values measured before the injection of the pH buffer and after the system reached equilibrium. The miRNA concentration was 10 nM, and the DNA nanoswitch concentration was 40 nM. Error bars represent the mean ± SD from *N* = 3 experiments.

To optimize TDNs reconfiguration transitions within the desired pH ranges, the TDNs were fine-tuned by varying the proportion of C-G·C^+^ and T-A·T triplets in the sequences: TDNsA with 20% or 30% T-A·T and TDNsB with 70% or 80% T-A·T. Each reporter unit (RA or RB) was labeled with a fluorophore (TAMRA), and each switch DNA (SA or SB) was labeled with a quencher (BHQ-2) to assess the binding behavior of the TDNs across different pH values. In Figure 2a, for the TDNsA (RA/SA hybrid) group, fluorescence recovery was observed at acidic pH, indicating the dissociation of SA and RA. This dissociation was attributed to the reconfiguration of SA into an intramolecular triplex structure. Moreover, the transition of TDNsA with 20% T-A·T occurred at a lower pH compared to the TDNsA with 30% T-A·T. This observation is consistent with previous reports suggesting that a lower percentage of T-A·T triplets (and higher C-G·C content) requires more H^+^ for protonation, thereby leading to a lower pH transition point.^3^ For the TDNsB (RB/SB hybrid) group, no fluorescence signals were observed at acidic pH values. As the pH increased, however, fluorescence signals gradually emerged, indicating the destabilization of the triplex structure and the dissociation of RB from SB. This behavior aligns with previous reports, which suggests that a triplex containing 70% T-A·T transitions at a lower pH compared to one with 80% T-A·T, as a higher percentage of T-A·T requires fewer protons (H^+^) for the transition.^62^ Furthermore, circular dichroism (CD) spectroscopy was employed to evaluate the triplex structure of the nanoswitches at varying pH levels. The results in Figure S6 showed that TDNsA with 30% T-A·T displayed a pronounced negative peak around 215 nm specifically at pH 5.0, whereas TDNsB with 70% T-A·T exhibited a similar negative peak at approximately 215 nm at both pH 5.0 and pH 7.0, confirming the presence of the triplex structure.^63^

Subsequently, the TDNs structures were functionalized with AuNPs to undergo the triplex–duplex transition in response to pH changes using the SPR platform, evaluating optimal reflectivity change. Figure 2b,c showed the real-time SPR sensorgrams for the optimization of TDNsA and TDNsB, respectively. The ΔRU fluctuations caused by the dissociation of AuNP-labeled reporter units at pH 8.3 or pH 5.0 are illustrated in Figure 2d,e. In Figure 2d, it is evident that only acidic pH conditions could trigger the transition of TDNsA, leading to the release of AuNPs@RA. In addition, TDNsA with 30% T-A·T exhibited a higher ΔRU compared to its 20% counterpart, attributed to its greater release efficiency and associated reflectivity changes in the pH 5.0 buffer. On the other hand, neither variant of TDNsB showed significant changes when exposed to the pH 5.0 buffer (Figure 2e). However, upon adjusting the buffer to pH 8.3, a marked decrease in the SPR responses was observed. This decrease is explained by the increased binding of AuNPs to TDNsB and the more efficient release of AuNPs@RB at pH 8.3, with the TDNsB variant containing 70% T-A·T demonstrating a greater response change compared to the 80% T-A·T variant.

Accordingly, TDNsA with 30% T-A·T and TDNsB with 70% T-A·T, which could successfully trigger the structure transition at specific pH values (Figure S7), were selected as the optimal combination of nanoswitches for the TDNs-SPR multiplex detection assay.

### Analytical Performance of Single Triplex DNA Nanoswitches for Detecting miRNAs

The SPR response, which correlates the refractive index changes as a result of mass changes/refractive index changes on the sensing interface, poses a challenge in distinguishing more than one target in a single SPR sensing channel. Motivated by the unique pH-induced reconfiguration features of triplex DNA nanostructures, two TDNs functionalized with AuNPs were employed as pH-responsive probes for dual miRNA sensing.

**Figure 3:**
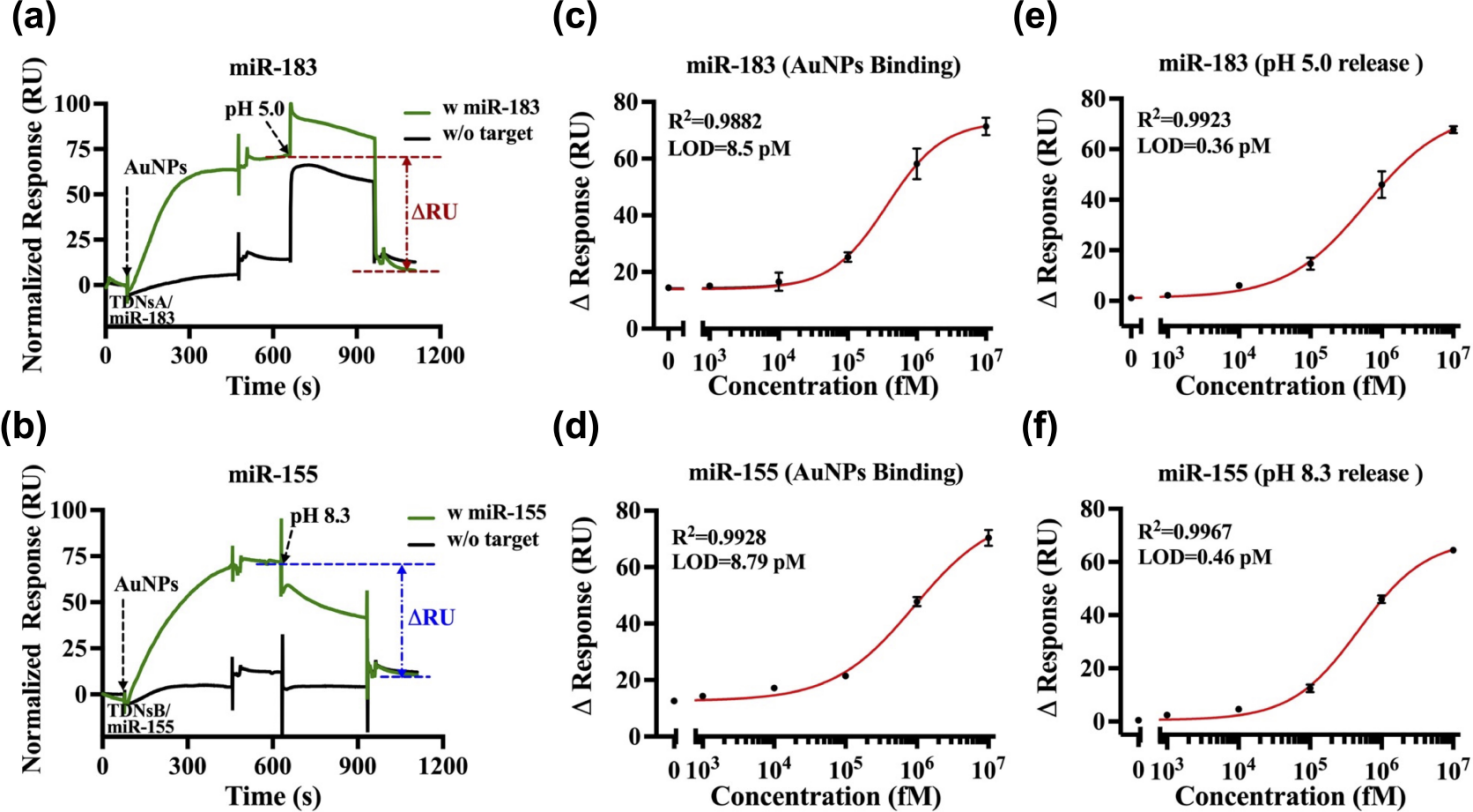
Analytical performance of singleplex miRNA detection using triplex DNA nanoswitches. (a, b) Feasibility tests for the detection of miR-183 and miR-155 using triplex DNA nanoswitch A and nanoswitch B, respectively. (c, d) Correlations between the logarithm of miR-183 and miR-155 concentrations and the response changes (ΔRU) caused by AuNPs binding. The fitted curve equation for panel (c) is , and for panel (d): . (e, f) Correlations between the logarithm of miR-183 and miR-155 concentrations and the response changes (ΔRU) resulting from the release of AuNP-labeled reporter units at pH 5.0 and pH 8.3, respectively. The fitted curve equation for panel (e) is ; for panel (f): . MiRNA concentrations tested were 0 pM, 1 pM, 10 pM, 100 pM, 1 nM, and 10 nM. The limits of detection (LODs) were calculated as the mean of the blank plus three times the standard deviation of the blank. Error bars represent the mean ± SD from *N* = 3 experiments.

First, the feasibilities of singleplex detection of target A (miR-183) and target B (miR-155) with the pH-responsive TDNsA and TDNsB, respectively, were assessed. The hybridization time interval for the formation of TDNs/miRNA hybrids was optimized, revealing that a 20 min incubation period at room temperature (pH 7.0) was sufficient to achieve a stable complex formation. (Figure S8). Subsequently, the TDNs/miRNA hybrids were injected into the SPR device and captured by the S9.6 antibody-immobilized interface. Following this, streptavidin-conjugated AuNPs (Strep-AuNPs), with a diameter of 40 nm, were introduced. Previous studies have demonstrated that 40 nm AuNPs provide optimal SPR biosensor enhancement for nucleic acids and protein detection.^64−67^ These nanoparticles are known to enhance the SPR response through coupling between the AuNPs plasmon and the surface plasmon wave.^68^ The results depicted in Figure 3a,b show a measurable increase in SPR response, indicating successful biotin–streptavidin interaction and the formation of the AuNPs@TDNs/miRNA complexes. However, following the injection of pH 5.0 and pH 8.3 releasing buffers, a noticeable decrease in response was observed.

Next, dose–response curves of miRNA at various concentrations were systematically evaluated and validated. Figure 3c,d show the change in SPR response (ΔRU) values associated with AuNPs binding, plotted against the logarithm of target concentrations. The limits of detection (LOD), defined by the International Union of Pure and Applied Chemistry (IUPAC) as three times the standard deviation of the blank, were calculated to be 8.50 pM for miR-183 and 8.79 pM for miR-155. This approach is consistent with previous studies.^69,70^ Moreover, Figure 3e,f demonstrate that in the absence of target miRNAs, the release of AuNPs@reporter unit (RA or RB), as indicated by the change in SPR response (ΔRU), is negligible at pH 5.0 for miR-183 detection and at pH 8.3 for miR-155 detection. As target concentrations increase, both ΔRU values progressively rise, with LODs determined to be 0.36 pM for miR-183 and 0.46 pM for miR-155. Furthermore, the calibration curves, which were fitted using a four-parameter logistic equation, exhibit very good correlations between the ΔRU values and the logarithm of target concentrations in the range of 1 pM to 10 nM. Notably, the LODs derived from AuNPs binding ΔRU were higher than those obtained from AuNPs releasing ΔRU. This discrepancy is likely attributable to the nonspecific adsorption of AuNPs onto the sensing chip, which can adversely affect the signal-to-noise ratio and thus diminish the sensitivity of the detection system.

Nonspecific adsorption of AuNPs on SPR platforms is a common issue,^43^ and several strategies have been developed to minimize this limitation. These include the incorporation of surfactants into the running buffer and the application of various antifouling agents to modify both AuNPs and the Au sensing interface.^71^ However, these methods can introduce additional complexity into the sensing process, and the effectiveness of antifouling measures may be limited. Previous studies by Luan et al.^72^ and Špringer et al.^65^ have addressed this issue by evaluating the AuNPs releasing response. They utilized restriction endonucleases to cleave the AuNPs bound to the SPR sensing surface or employed the DNA strand displacement reactions to trigger the release of AuNPs, thereby evaluating the extent of nonspecific binding of the nanoparticles and the impact on the transducing signal. Our findings are consistent with these reports, as the releasing response of AuNPs is guided by the pH-responsive TDNs at specific pHs, instead of relying on the binding response of AuNPs. This approach minimizes the background signals and further enhances the detection sensitivity for dual miRNA detection.

### Dual Detection of miRNAs Using Triplex DNA Nanoswitches in a Single Run on the SPR Platform

**Figure 4:**
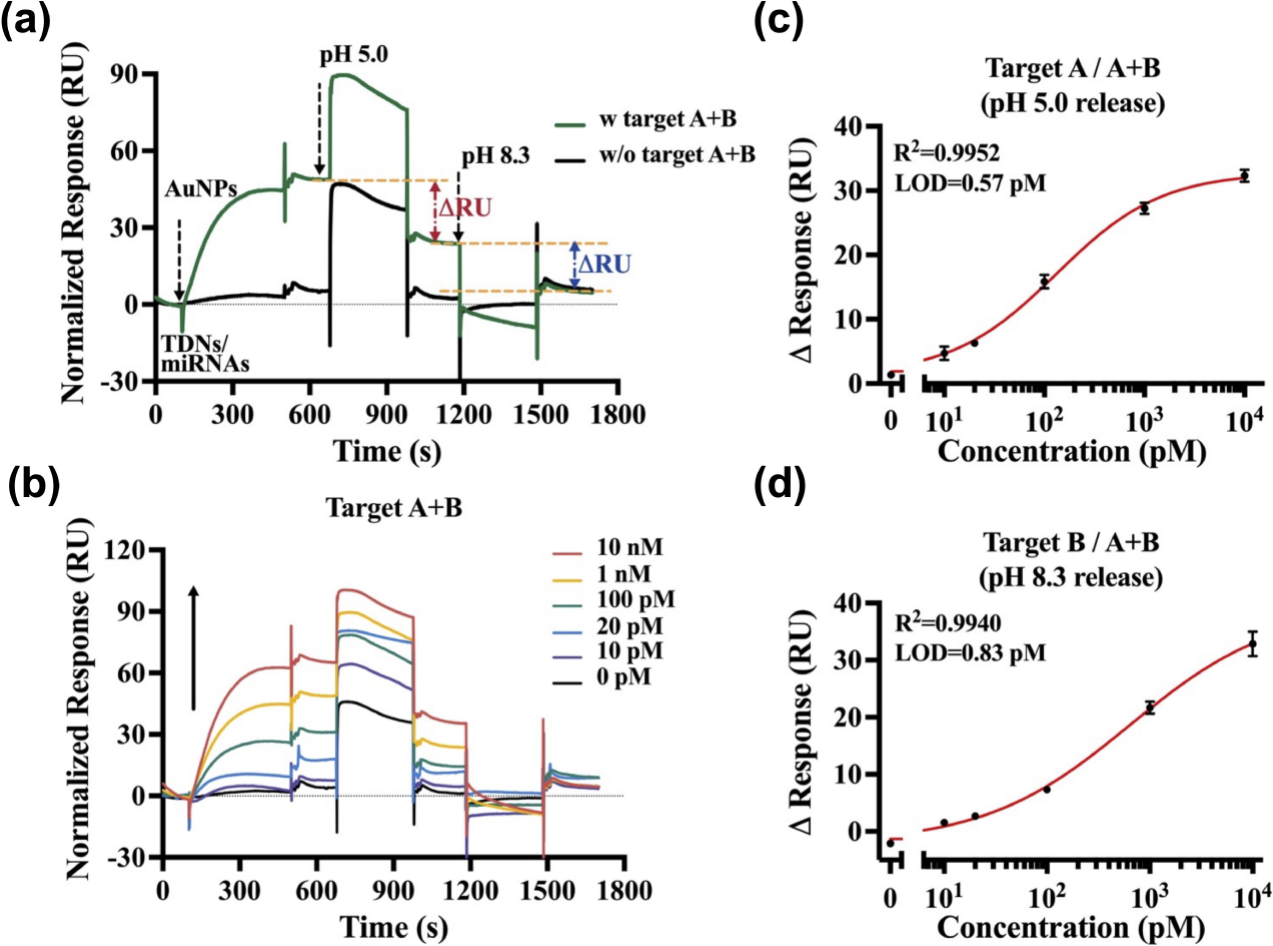
Dual detection of miRNAs using triplex DNA nanoswitches in a single run on the SPR platform. (a) Feasibility assessment for the detection of miRNAs utilizing triplex DNA nanoswitches. (b) Sensorgrams illustrating the dual detection of miRNAs across a concentration range from 10 pM to 10 nM. (c) Calibration curve depicting the response changes (ΔRU) attributed to the release of AuNPs-labeled reporter unit A (AuNPs@RA) at pH 5.0 versus the logarithm of miR-183 (target A) concentration. The fitted curve equation for panel (c) is . (d) Calibration curve showing the response changes (ΔRU) resulting from the release of AuNPs-labeled reporter unit B (AuNPs@RB) at pH 8.3 versus the logarithm of miR-155 (target B) concentration. The fitted curve equation for panel (d) is . Target miRNAs were used at concentrations of 0 pM, 10 pM, 20 pM, 100 pM, 1 nM, and 10 nM. Limits of detection (LODs) were determined as blank plus three times the standard deviation of the blank. Error bars represent the standard deviation from *N* = 3 experiments.

To evaluate the feasibility of dual detection of miRNAs, equal amounts of TDNsA/target A (miR-183) and TDNsB/target B (miR-155) were mixed in one pot. Figure 4a demonstrates that in the absence of miRNAs, no significant changes in the SPR responses after the sequential injection of pH 5.0 and pH 8.3 buffers were observed. Upon the addition of a mixture of target miRNAs, two significant response changes were observed at the corresponding pHs, with values comparable to those obtained in single detection assays, suggesting the potential application of the platform for dual target detection. Figure 4b illustrates the overall SPR sensorgrams for dual detection of miRNAs in the concentration range of 10 pM to 10 nM. As the injection of Strep-AuNPs, the SPR response intensified with the increasing concentration of the miRNAs. After the pH 5.0 and pH 8.3 buffer flowed along the surface sequentially, two significant decreases in SPR response were observed. By plotting the changes in response (ΔRU) resulting from the release of AuNPs-labeled reporter units at pH 5.0 and pH 8.3 against the logarithmic concentrations of miR-183 and miR-155, correlation coefficients of 0.995 and 0.994 were achieved, respectively. (Figure 4c,d). The LODs were calculated to be 0.57 pM and 0.83 pM, showing only small variations compared to singleplex detection.

To the best of our knowledge, achieving the simultaneous detection of multiple targets within a single channel using a single run on an SPR biosensor is an analytical challenge. By employing TDNs as pH-responsive probes, the proposed assay demonstrates the capability for duplex detection through a straightforward pH-mediated transition of the DNA sensing probes. This approach utilizes the pH-induced reconfiguration of the probes to enable the detection of multiple targets. When integrated with microfluidic devices, it holds promise for application in multiplex detection. The rapid triplex-to-duplex transition, triggered by pH changes, allows the detection of two miRNAs in an hour without amplification.^62^ While some biosensors may show higher sensitivity compared to our method, they often involve multiple amplification steps, which are time-consuming and complex.^45,73−77^ Our TDNs-SPR assay, characterized by high sensitivity, a short assay time, a straightforward experimental process, demonstrates comparable or superior performance to existing miRNA biosensors employing SPR platforms (Table S3).

### Selectivity and Cross-Reactivity Test of the Triplex DNA Nanoswitches

**Figure 5:**
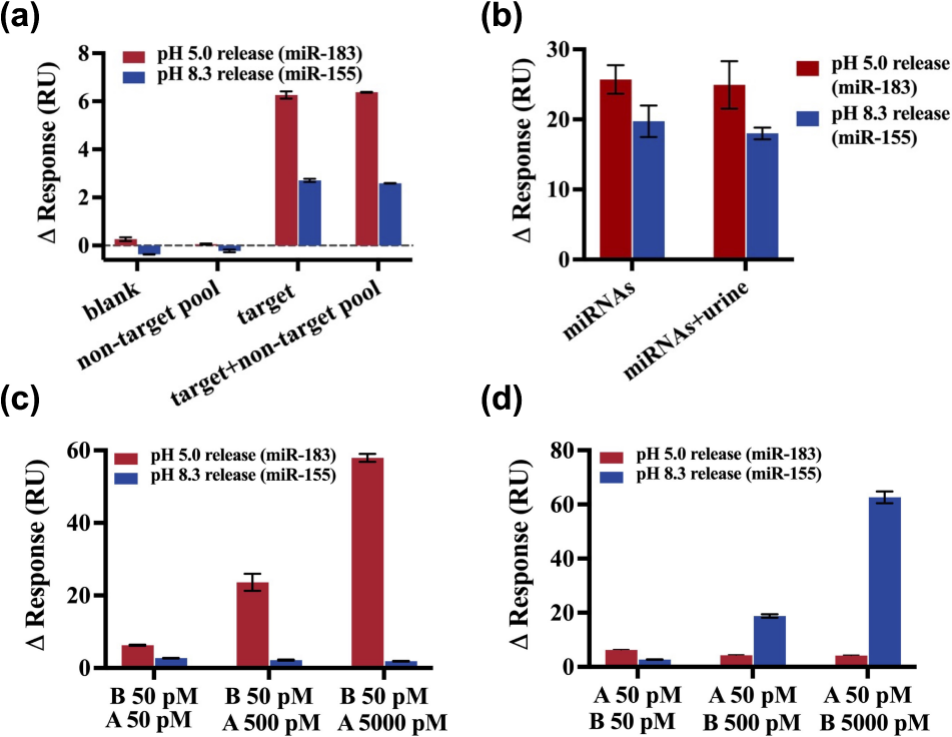
Selectivity and cross-reactivity tests of the triplex DNA nanoswitches. (a) Selectivity test in the presence of high concentration of a nonspecific miRNAs pool (miR-141, miR-21, miR-205, miR-210) toward the detection of low concentrations of specific target miR-183 and miR-155. Each nonspecific miRNA was 250 pM and the specific miRNA was 25 pM. (b) Recovery test by spiking miRNAs into artificial urine. Each target miRNA was spiked at 1 nM into the buffer and artificial urine, respectively. (c, d) Cross-reactivity study by measuring the one target miRNA (50 pM) in the presence of different concentrations of the other target miRNA (50 pM, 500 pM, 5000 pM). The SPR response change (ΔRU) at pH 5.0 corresponds to the detection of target A (miR-183), while the SPR response change (ΔRU) at pH 8.3 corresponds to the detection of target B (miR-155). Error bars represent the standard deviation from *N* = 3 experiments.

Due to the high sequence homology among miRNAs, it was imperative to assess the assay’s capability to discriminate between closely related miRNAs. To address this issue, we selected cancer-associated miRNAs^20^ and members of the miR-183 family, exhibiting significant sequence similarities, for a rigorous selectivity evaluation. PAGE analysis (Figure S9) revealed that only specific miRNAs could bind to their corresponding switch DNA. Moreover, the four cancer-associated miRNAs (miR-141, miR-21, miR-205, miR-210) were mixed at high concentrations (250 pM) to create a nonspecific miRNAs pool to evaluate the selectivity performance on our proposed SPR platform. The results depicted in Figure 5a reveal similar response change (ΔRU) between the group with target miRNAs and the group with target miRNAs mixed with nonspecific miRNAs pool. These results indicate that high concentrations of the foreign miRNAs do not interfere with the detection of low concentrations of the target miRNAs.

Furthermore, to assess the detection performance in a complex biological matrix, target miRNAs were spiked into artificial urine (Surine negative urine control, Cerilliant, USA). As shown in Figure 5b, the detection results in the artificial urine closely matched those obtained in the buffer system, with a recovery rate exceeding 90%. These findings suggest the method’s robust applicability for potential clinical sample analysis.

For the cross-reactivity test, one target miRNA was maintained at a fixed concentration of 50 pM, while the other miRNA was introduced at varying concentrations (50 pM, 500 pM, 5000 pM). Figure 5c,d reveal that as dual miRNAs coexist in the sample, only when the concentration of one miRNA is 100 times higher than the other, such as 5000 pM versus 50 pM, a minute effect on detecting lower concentration miRNA may occur. This may originate from the occupation of antibody binding sites associated with the sensing interface by a high-concentration miRNA, thus perturbing the binding of the low-concentration of other miRNAs. However, the abundance of miRNAs in biological samples is far lower, therefore this perturbance may be considered negligible.

Taken together, the results demonstrate high specificity and negligible cross-reactivity for dual miRNAs detection by the triplex DNA nanoswitch platform, suggesting its potential application for real sample analysis with multiple nontargeted miRNAs.

### Real Sample Analysis Using Urine Samples from Bladder Cancer Patients

**Figure 6:**
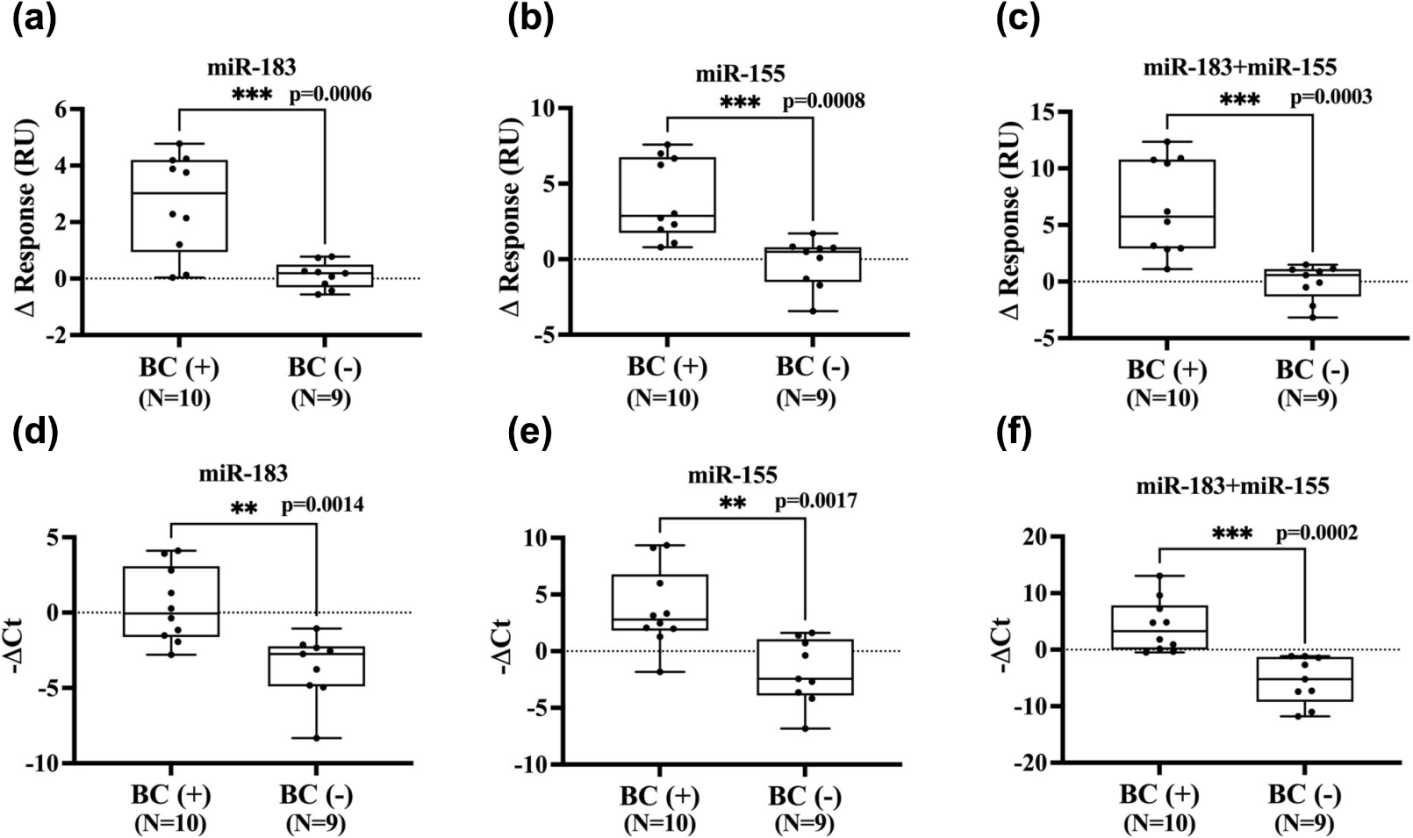
Box plots for urine miRNAs expression levels of bladder cancer (BC) patients and healthy controls. (a, b) SPR response change (ΔRU) for miR-183 and miR-155 detection using our SPR assay. (c) Summed response change (ΔRU) values of miR-183 and miR-155 in each urine sample. (d, e) Relative expression of miR-183 and miR-155 using qRT-PCR. miRNA levels were determined from the cycle threshold (Ct) values, and relative miRNA levels (-ΔCt) were calculated using the equation: -ΔCt = −(Ct value of target miRNA-Ct value of RNU6B). (f) Summed relative miRNA Ct (-ΔCt) values for miR-183 and miR-155 in each urine sample. The statistical difference between the two groups was determined using a two-tailed Student’s *t* test, with p-values of >0.05(ns), ≤ 0.05(*), ≤ 0.01(**), ≤ 0.001(***), ≤ 0.0001(****) at a 95% confidence interval.

To evaluate the capability for real sample analysis, urine samples from 10 BC patients and 9 healthy individuals were selected. Total miRNAs were extracted from the samples using a commercial miRNA purification kit (miRNeasy Serum/Plasma Kit) according to the manufacturer’s recommended protocol. The extracted miRNA samples were incubated with two distinct TDNs probes designed to selectively detect miR-183 and miR-155, respectively. As shown in Figure 6a,b, ΔRU at pH 5.0 for miR-183 and at pH 8.3 for miR-155 detection reveal significantly elevated values in BC patients compared to healthy individuals, with p-values of 0.0006 and 0.0008, respectively. Interestingly, summing the two ΔRU values for each urine sample further accentuated the distinction between BC patients and healthy individuals (Figure 6c).

Furthermore, a commercial qRT-PCR assay was employed for validation. As illustrated in Figure 6d,e, the results obtained from the TDNs-SPR assay closely aligned with those acquired from the qRT-PCR assay. The results are in agreement with previous studies that associate overexpression of miR-183 and miR-155 in urine with BC diagnosis,^23,26^ indicating the feasibility of our developed biosensor for dual miRNA detection in clinical samples.

In 2016, researchers conducted a statistical analysis of various studies on the use of miRNAs for BC diagnosis. The analysis revealed that using a single miRNA biomarker achieved a sensitivity of approximately 72% and a specificity of 77%. In contrast, the application of multiple miRNAs for diagnosis enhanced the sensitivity to approximately 82%.^78^ This highlights the importance of developing a nucleic acid biosensor with multiplexing capability. To address this need, Cheng et al. demonstrated the use of exponential amplification methods coupled with Au nanoparticles and lateral flow nucleic acid biosensors to significantly improve the detection of BC-specific miRNAs.^79^ Xu et al. used magnetic quantum dot microbeads and catalytic hairpin assembly for multiplex BC-specific miRNAs detection.^80^ Zhang et al. integrated Au nanoparticles with different fluorescence carbon dots and utilized DNAzyme as a signal amplifier for dual BC-related exosome miRNA detection.^81^ The comparison is presented in Table S3. The proposed TDNs-SPR sensing platform presents a notable advantage by obviating the necessity for conventional nucleic acid amplification techniques or intricate sample preparation procedures. Additionally, the reaction operates at room temperature and is completed within an hour, substantially reducing the reaction time. This approach leverages the dynamic reconfiguration of DNA nanostructures, offering an efficient and simplified strategy for miRNA detection.

Moreover, it should be noted that the flexibility and high capacity of the TDNs-SPR sensing platform have the potential to significantly improve assay sensitivity and facilitate effective multiplexed detection. By conjugating AuNPs directly to the pH-responsive triplex DNA switches, rather than to smaller reporter DNA strands, the platform capitalizes on enhancing the amplified SPR response of the switch DNA. This design strategically enhances sensitivity by leveraging the reflectivity changes produced during the pH-triggered release of the AuNPs@switch DNA complex. Moreover, the plasmonic coupling interaction between the nanoparticles and the surface plasmon wave can be increased due to a short interaction distance, thereby enhancing sensitivity. Furthermore, the use of a multichannel SPR device for operating the dual triplex DNA nanoswitch platform holds great promise for highly sensitive specific multiplexed miRNA analysis.

## Conclusion

The study introduced a versatile sensing platform for the stepwise surface plasmon resonance (SPR) detection of two different miRNAs using pH-responsive triplex DNA nanoswitches as sensing probes (TDNs-SPR assay). This sensing platform was applied to detect the miR-183 and miR-155 associated with bladder cancer. The sensing platform is based on the assembly of two triplex probes composed of varied contents of C-G·C^+^/T-A·T pH-responsive triplet sequences anchored to the SPR sensing interface by specific DNA/miRNA duplexes linked to the S9.6 antibody-modified surface. The sensing probes are functionalized with biotinylated reporter nucleic acid units modified with AuNPs-labeled streptavidin. The pH-induced displacement of the reporter units was achieved through the stepwise reconfiguration of the triplex nanoswitch probes. For example, at pH 5.0, the reconfiguration into the C-G·C^+^ triplex facilitated the detection of miR-183, while at pH 8.3, the dissociation of the T-A·T triplex enabled the sensing of miR-155. These sequential reconfigurations and releases of reporter units were transduced into distinct changes in SPR reflectivity, thereby indicating the functional response of the device. The sensing platform demonstrated high sensitivities reflected by detection limits of 0.57 pM and 0.83 pM for miR-183 and miR-155, respectively, and high specificity originating from the selective association of the respective DNA/miRNAs to the antibody-modified surface. The sensing platform was successfully applied to detect the bladder cancer biomarkers miR-183 and miR-155 in urine samples of bladder cancer-carrying individuals and control healthy individuals. The analytical results were comparable to qPCR analyses of the same samples. Moreover, this platform can be adapted to detect a broad spectrum of target miRNAs. These features position it as a promising tool for the noninvasive, early detection of bladder cancer and other miRNA-related diseases.

## Materials and Methods

### Construction of Triplex DNA Nanoswitches

The triplex DNA nanoswitch (TDNs) was constructed by combining switch DNA with reporter DNA. To form the hairpin structure, the switch DNA was initially denatured at 95 °C for 5 min, followed by gradual cooling to 25 °C over the course of 2 h. Subsequently, the switch DNA (40 nM) was incubated with the corresponding biotin-labeled reporter DNA (200 nM) at room temperature for 1 h in 10 mM HEPES buffer (15 mM MgCl_2_, 80 mM NaCl, pH 7.0). The assembled TDNs were stored at −20 °C for future use.

### Preparation of S9.6 Antibody-Modified CM5 Sensing Chip

The S9.6 antibody was immobilized on a CM5 chip using the amine coupling method according to the manufacturer’s recommended protocol. The chip was activated by the EDC/NHS mixture, followed by an injection of S9.6 antibody (30 μg/mL) in 10 mM sodium acetate buffer (pH 4.5). Unreacted sites were subsequently blocked with 1 M ethanolamine-HCl. All surface plasmon resonance (SPR) experiments were performed in 10 mM HEPES running buffer (15 mM MgCl_2_, 80 mM NaCl, at pH 7.0) at a flow rate of 20 μL/min. Samples were flowed through both a reference channel and an S9.6 antibody-immobilized sensing channel. To account for temperature fluctuations, nonspecific binding, and bulk refractive index changes, the final response was normalized by subtracting the response units (RU) of the reference channel from those of the sensing channel. After each sample injection, the S9.6 antibody-immobilized chip was regenerated with 10 mM glycine-HCl (pH 1.7) and 2 M MgCl_2_ for 15 s each, followed by rinsing with a running buffer until equilibration was achieved. The chip was then stored in the running buffer at 4 °C for future use.

### TDNs-SPR Assay for Dual-Detection of miRNAs

After hybridizing the miRNAs with the corresponding TDNs at room temperature for 20 min, the TDNs/miRNA mixture (A+B) was introduced into the SPR system, where it was captured by the immobilized S9.6 antibody on the chip for 240 s. Subsequently, streptavidin-AuNPs (Strep-AuNPs) with an optical density (OD) of 0.056 (1:180 dilution) were then flowed over the surface for 400 s, allowing them to bind to the biotin-reporter unit on the TDNs/miRNA complexes, thereby enhancing the SPR response (AuNPs@TDNs/miRNA). To release the AuNPs@reporter units from the complexes, pH 5.0 and pH 8.3 buffers were sequentially passed over the surface for 300 s each, with a 120 s rinse using a running buffer between pH transitions to reach equilibrium. The SPR response was recorded in resonance units (RU), and the difference in SPR response (ΔRU) was calculated by comparing the values before and after the sample was injected and reached equilibrium. The pH 5.0 and pH 8.3 buffers were prepared using 20 mM HEPES (containing 5 mM MgCl_2_, 80 mM NaCl) and adjusted to the respective pH levels with HCl and NaOH.

For the evaluation of analytical performance, the change in SPR response (ΔRU) was plotted against the logarithmic scale of varying miRNA concentrations. Calibration curves were generated using GraphPad Prism software (Boston, MA, USA) and modeled with a four-parameter logistic equation:
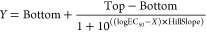


where Y represents the ΔRU, X corresponds to the logarithm of the target concentration, Top signifies the maximum ΔRU, and Bottom denotes the minimum ΔRU in the absence of the target.

### Polyacrylamide Gel Electrophoresis Analysis (PAGE)

To determine the optimal hybridization time for the formation of switch DNA/miRNA hybrids and evaluate the selectivity of the TDNs, 15% PAGE was conducted in 0.5X TBE buffer (50 mM Tris-base, 41.5 mM Boric acid, 0.5 mM EDTA) under a constant voltage of 80 V for 100 min at room temperature. For the binding specificity analysis of the S9.6 antibody toward switch DNA/miRNA hybrids, an electrophoretic mobility shift assay (EMSA) was employed. Switch DNA (200 nM) was hybridized with DNA and RNA targets (200 nM each) for 20 min, followed by incubation with S9.6 antibody (80 μg/mL) for 30 min at room temperature. A 6% PAGE was prerun at a constant voltage of 100 V for 30 min in 0.5X TBE buffer at room temperature, after which the samples were loaded and run at 80 V for 60 min. The gels were subsequently stained with SYBR Gold for 10 min, imaged using a gel imaging system (Biorad, Hercules, CA, USA), and analyzed via Image Lab software (Biorad, Hercules, CA, USA).

### Optimization of the pH-Responsive TDNs by Fluorescence Assay

The reporter DNA was labeled with a pH-insensitive fluorophore (Carboxytetramethylrhodamine, TAMRA), while the switch DNA was labeled with a quencher (Black Hole Quencher 2, BHQ-2). Switch DNA (200 nM) was hybridized with the reporter DNA (200 nM) at room temperature for 20 min in various pH buffers (pH 4.5, 5.0, 6.0, 7.0, 8.0, 8.5, 9.0). Following hybridization, 20 μL of the reaction mixture was transferred to a 384-well plate, and fluorescence intensity was measured with excitation at 557 nm and emission at 583 nm (*λ*_*ex*_*=*557 nm, *λ*_*em*_ = 583 nm). Relative fluorescence was calculated using the formula , where F represents the sample fluorescence, and *F*_min_ and *F*_max_ denote the minimum and maximum fluorescence values for each TDNs group, respectively.

### Detection of miRNAs in Urine Samples Using Proposed SPR Assay

Clinical urine samples were provided by Dr. Cheng-Che Chen from Taichung Veterans General Hospital (Taichung, Taiwan). The samples comprised 10 from bladder cancer patients and 9 from healthy individuals. The analysis of these clinical samples was conducted following approval from the Institutional Review Board (IRB) of Taichung Veterans General Hospital (IRB no. CE21454B–2). Written informed consent was obtained from all participants, and the urine samples were collected by a trained professional using sterile techniques. Following collection, 7.5 mL of urine was centrifuged at 12000 × g for 15 min at 4 °C. The supernatant was discarded, and 1 mL of the remaining sediment was stored at −80 °C until RNA extraction. MiRNAs were extracted using the miRNeasy Serum/Plasma kit (Qiagen, Hilden, Germany) according to the manufacturer’s recommended instructions. Briefly, 300 μL of the urine sample was lysed in QIAzol, followed by chloroform extraction. After centrifugation, the RNA-containing aqueous phase was mixed with ethanol, loaded into a spin column, and finally eluted with 14 μL of RNase-free water. For real sample analysis, the extracted RNA was mixed with two triplex DNA switches to obtain a 120 μL solution, which was then injected into the SPR system for dual target miRNA detection.

### Cross-Validation of the SPR Results for Clinical Samples with qRT-PCR

The quantitative real-time polymerase chain reaction (qRT-PCR) analysis was performed using the TaqMan MicroRNA Assays Kit (Thermo Fisher Scientific, Rockford, IL, USA) and TaqMan MicroRNA Reverse Transcription Kit (Thermo Fisher Scientific, Rockford, IL, USA), adhering to the manufacturer’s protocols. To quantify miR-183, miR-155, and RNU6B (an endogenous control) in urine samples, a 5 μL aliquot of extracted RNA was mixed with reverse transcription primer and master mix, including dNTPs, reverse transcriptase, reverse transcription buffer, and RNase inhibitor. The reverse transcription reaction was conducted at 16 °C for 30 min, 42 °C for 30 min, and 85 °C for 5 min. Subsequently, the RT product was combined with TaqMan microRNA probes and TaqMan Universal Master Mix II to prepare a 20 μL reaction mixture. The qRT-PCR was executed with an initial denaturation at 95 °C for 10 min, followed by 45 cycles of amplification at 95 °C for 15 s and 60 °C for 60 s. miRNA levels were determined from the cycle threshold (Ct) values, and relative miRNA levels (-ΔCt) were calculated using the equation: -ΔCt = −(Ct value of target miRNA-Ct value of RNU6B).^82^ This method validation ensures the accuracy of the SPR-based miRNA detection and confirms its effectiveness in clinical sample analysis.

## Supporting Information Available

Materials and methods; The oligonucleotide sequences utilized for the experimental optimization; Binding kinetics of S9.6 antibody with DNA/miRNA hybrid switches; Comparison of various analytical methods for miRNA detection; Performance of the pH-insensitive fluorophore; Valuation of the pH stability of target/switch DNA hybrids; Binding stability of S9.6 antibody toward switch DNA/miRNA hybrids; Optimization of MgCl_2_ concentration in the SPR running buffer; Regeneration efficiency of the S9.6 antibody-immobilized SPR CM5 chip; Circular dichroism (CD) spectroscopy method for the analysis of triplex DNA nanoswitches under different pH conditions; pH-specificity of TDNs for miRNA analysis using the SPR platform; Optimization of target miRNAs and TDNs hybridization time using PAGE; Specificity evaluation of TDNs via PAGE; Analysis of microRNA levels in real urine samples determined by SPR and qRT-PCR (PDF)
