# Supplementary material for: pH-Responsive Triplex DNA Nanoswitches: Surface Plasmon Resonance Platform for Bladder Cancer-Associated microRNAs
Source: ACS Nano. 2025 Feb 12;19(7):7140–53. doi: 10.1021/acsnano.4c16396 (PMC11867022; doi:10.1021/acsnano.4c16396)
Supplement: Supplementary file 1 — nn4c16396_si_001.pdf [file nn4c16396_si_001.pdf]

Supporting Information

pH-Responsive Triplex DNA Nanoswitches: Surface Plasmon Resonance Platform for Bladder Cancer-Associated microRNAs

Pei-Ying Lin,[a+] Ying-Feng Chang,[a,b,c+] Cheng-Che Chen,[a,d,e+] Li-Chen Su,[f,g] Itamar Willner,[h*] Ja-an Annie Ho[a,i,j,k*]

[a]BioAnalytical Chemistry and Nanobiomedicine Laboratory, Department of Biochemical Science and Technology, National Taiwan University, Taipei 10617, Taiwan
[b]Artificial Intelligence Research Center, Chang Gung University, Taoyuan 33302, Taiwan
[c]Department of Gastroenterology and Hepatology, New Taipei Municipal Tucheng Hospital (Built and Operated by Chang Gung Medical Foundation), New Taipei 23652, Taiwan
[d]Department of Urology, Taichung Veterans General Hospital, Taichung 40705, Taiwan
[e]Department of Medicine and Nursing, Hungkuang University, Taichung 43304, Taiwan
[f]Organic Electronics Research Center, Ming Chi University of Technology, New Taipei 243303, Taiwan
[g]General Education Center, Ming Chi University of Technology, New Taipei 243303, Taiwan
[h]Institute of Chemistry, The Hebrew University of Jerusalem, Jerusalem 91904, Israel
[i]Department of Chemistry, National Taiwan University, Taipei 10617, Taiwan
[j]Center for Emerging Materials and Advanced Devices, National Taiwan University, Taipei 10617, Taiwan
[k]Center for Biotechnology, National Taiwan University, Taipei 10617, Taiwan
*Correspondence : itamar.willner@mail.huji.ac.il, jaho@ntu.edu.tw

Manuscript submitted to *ACS Nano*

[+] These authors contributed equally to this work.

*Correspondence: Prof. Itamar Willner (e-mail: itamar.willner@mail.huji.ac.il), Institute of Chemistry, The Hebrew University of Jerusalem, Jerusalem 91904, Israel.

*Correspondence: Prof. Ja-an Annie Ho (e-mail: jaho@ntu.edu.tw), BioAnalytical Chemistry and Nanobiomedicine Laboratory, Department of Biochemical Science and Technology/ Department of Chemistry, National Taiwan University, Taipei 10617, Taiwan. Tel: +886-2-33664438.

TABLE OF CONTENTS

Materials and Methods …………………………………………………………………..…………………….......S2

Supplemental Tables

Table S1. The oligonucleotide sequences utilized for the experimental optimization ……….………….......S4

Table S2. Binding kinetics of S9.6 antibody with DNA/miRNA hybrid switches ……….…………………....S5

Table S3. Comparison of various analytical methods for miRNA detection.………………………….........S6

Supplemental Figures

Figure S1. Performance of pH-insensitive fluorophore (TAMRA)..……………………………….…………..S7

Figure S2. Evaluation of the pH stability of target/switch DNA hybrids.…………………………..…..…S8

Figure S3. Binding stability of S9.6 antibody toward switch DNA/miRNA hybrids.……..……………..……S9

Figure S4. Optimization of $MgCl_2$ concentration in the SPR running buffer. …………………………..…...S10

Figure S5. Regeneration efficiency of the S9.6 antibody-immobilized SPR CM5 chip. ………………..…S11

Figure S6. Circular dichroism (CD) spectroscopy method for the analysis of triplex DNA nanoswitches under different pH conditions. ……………………………………………………...………………………..S12

Figure S7. pH-specificity of TDNs for miRNA analysis using the SPR platform ……………………….......S13

Figure S8. Optimization of target miRNAs and TDNs hybridization time using PAGE. …………………...S14

Figure S9. Specificity evaluation of TDNs via PAGE ……………………………………………….….…..…S15

Figure S10. Analysis of microRNA levels in real urine samples determined by SPR and qRT-PCR…..S16

References…………………………………………………………………………………...…………….……S17

MATERIALS AND METHODS

Materials

Sodium chloride (NaCl), 4-(2-hydroxyethyl)-1-piperazineethanesulfonic acid (HEPES), and magnesium chloride hexahydrate ($MgCl_2{\cdot}6H_2O$) were purchased from Sigma-Aldrich (St. Louis, MO, USA). Polyacrylamide (29:1), tetramethylethylenediamine (TEMED), and ammonium persulfate (APS) were obtained from MDBio Inc. (Taipei, Taiwan). Mouse anti-DNA-RNA hybrid antibody (S9.6 antibody) was acquired from Kerafast (Boston, MA, USA). Streptavidin-coated AuNPs (40 nm, 10 OD) were purchased from Cytodiagnostics (Burlington, ON, Canada).

Materials for SPR experiments, including 1-ethyl-3-(3-dimethylaminopropyl)-carbodiimide (EDC), N-hydroxysuccinimide (NHS), 1 M ethanolamine-HCl (pH 8.5), 10 mM sodium acetate (pH 4.5), 10 mM glycine-HCl (pH 1.7), and Series S CM5 sensing chips, were obtained from Cytiva (Marlborough, MA, USA). The miRNeasy Serum/Plasma Kit was purchased from Qiagen (Hilden, Germany). SYBR Gold, GeneRuler ultra-low range DNA ladder, TaqMan microRNA assays kit, TaqMan microRNA reverse transcription kit, and TaqMan universal master mix II (no UNG) were purchased from Thermo Fisher Scientific (Rockford, IL, USA). All solutions were prepared in Milli-Q water (18.2 MΩ·cm).

Oligonucleotides were purchased from Integrated DNA Technologies (Coralville, IA, USA), while fluorophore/quencher-modified oligonucleotides were acquired from Protech Technology Enterprise (Taipei, Taiwan). The main sequences are included in the main text, while the other sequences used for optimizing the experiment are listed in **Table S1**.

Apparatus

Native polyacrylamide gel electrophoresis (PAGE) was performed using the Mini-Protean Tetra Cell, with the gel visualized on a ChemiDoc™ Imaging System (Bio-Rad, Hercules, CA, USA). SPR experiments were conducted on a Biacore T200 instrument (Cytiva, Burlington, MA, USA). Fluorescence measurements were obtained using a Varioskan LUX Multimode Microplate Reader (Thermo Fisher Scientific, Rockford, IL, USA). miRNA qPCR analysis was carried out on a Roche LightCycler® 96 Instrument (Basel, Switzerland).

.

Methods

Single-Cycle Kinetic Analysis with SPR

The binding kinetics of DNA/miRNA hybrid switches to the S9.6 antibody were investigated using an SPR single-cycle kinetics assay, which obviated the need for regeneration between sample injections. The switch DNA/miRNA hybrids (0.32 nM, 1.6 nM, 8 nM, 40 nM, 200 nM) were sequentially flowed over the S9.6 antibody-immobilized chip for 120 s, followed by a 60-second dissociation phase at a flow rate of 30 μL/min. The binding kinetics were analyzed by fitting the sensorgrams to the Langmuir 1:1 binding model using Biacore T200 Evaluation software.

Circular Dichroism (CD) Measurement

Circular dichroism (CD) spectroscopy was performed using a Jasco model J-815 spectropolarimeter (JASCO International, Tokyo, Japan). Reporter DNA and switch DNA (each at a concentration of 2 μM) were hybridized in a 20 mM phosphate buffer solution containing 80 mM NaCl and 5 mM $MgCl_2$ across various pH levels for 30 min at room temperature. Subsequently, a 500 μL sample was analyzed using a quartz cuvette. CD spectra were recorded in the wavelength range of 205 to 300 nm with a 1 nm bandwidth and a scanning speed of 50 nm per minute at room temperature.

SUPPPLEMENTAL TABLES AND FIGURES

Table S1. The oligonucleotide sequences utilized for the experimental optimization.

**Name**	**Sequence (5'-3')**
**SPR measurement**	
Switch A (20% TAT)	**AGTGAATTCTACCAGTGCCATA**GAGGAGGGGAGGGGAGGGGAGGTTTACCTCCCCTCCCCTCCCCTCCCTTTGCCTCCCCTCCCCTCCCCTCCGT
Reporter B (80% TAT)	TTCTTTTCTTTTCTTTTCTTTTT-Biotin
Switch B (80% TAT)	AAGAAAAGAAAAGAAAAGAACACCTTCTTTTCTTTTCTTTTCTTAG**ACCCCTATCACGATTAGCATTAA**
**Fluorescence measurement**	
Target A-F	TATGGCACTGGTAGAATTCACT-TAMARA
Switch A-Q	BHQ2**-**<br>**AGTGAATTCTACCAGTGCCATA**GAGGAGAGGAGAGGAGGGGAGGTTTACCTCCCCTCCTCTCCTCTCCCTTTGCCTCTCCTCTCCTCCCCTCCGT
Target B-F	TAMRA-TTAATGCTAATCGTGATAGGGGT
Switch B-Q	AAGAAAAGAGAAGAAGAGAACACCTTCTCTTCTTCTCTTTTCTTAG**ACCCCTATCACGATTAGCATTAA-**BHQ2
Reporter A-F	TAMRA-ACGGAGGGGAGG
Switch A (20% TAT)-Q	ATAGAGGAGGGGAGGGGAGGGGAGGTTTACCTCCCCTCCCCTCCCCTCCCTTTGCCTCCCCTCCCCTCCCCTCCGT-BHQ2
Switch A (30% TAT)-Q	ATAGAGGAGAGGAGAGGAGGGGAGGTTTACCTCCCCTCCTCTCCTCTCCCTTTGCCTCTCCTCTCCTCCCCTCCGT-BHQ2
Reporter B (70% TAT)-F	TAMRA-TTCTTTTCTCTTCTTCTCTT
Switch B (70% TAT)-Q	BHQ2-AAGAAAAGAGAAGAAGAGAACACCTTCTCTTCTTCTCTTTTCTTAGAC
Reporter B (80% TAT)-F	TAMRA-TTCTTTTCTTTTCTTTTCTT
Switch B (80% TAT)-Q	BHQ2-AAGAAAAGAAAAGAAAAGAACACCTTCTTTTCTTTTCTTTTCTTAGAC

Note： The bold letters represent the sequences complementary to the corresponding miRNAs, while the underlined bases indicate the triplex-forming sequences.

Table S2. Binding kinetics of S9.6 antibody toward DNA/miRNA hybrid switches.

Sample	k_a (1/Ms)	k_d (1/s)	K_D (M)	Chi² (RU²)
**S9.6 Ab-miR-183/Switch A**	5.99×10^{5}	1.74×10^{-4}	2.91×10^{-10}	24.6
**S9.6 Ab-miR-155/Switch B**	3.26×10^{5}	4.69×10^{-5}	1.44×10^{-10}	22.5

k_a: the association rate constant (M^{-1} s^{-1}), k_d: the dissociation rate constant (s^{-1}), K_D: The equilibrium dissociation constant of k_d / k_a (M).

Table S3. Comparison of various analytical methods for miRNA detection.

Platform	Amplification methods	LOD	Reaction temperature	Assay time	MiRNAs	Sample type	Reference
Fluorescence	CRISPR-Cas13a and rolling circle amplification	0.2~0.6 pM	37 °C	75 min	miR21, 141, Let7b	Serum (Prostate Cancer)	[1]
Fluorescence imaging	Quantum dot microbeads with CHA and enzyme mediated DNA polymerization	4 fM	37 °C	180 min	miR-96, 200a, 135b	Serum (Bladder cancer)	[2]
Fluorescence	AuNP modified with carbon dot-labeled substrates and DNAzyme	10 fM	37 °C	180 min	miR-133b, 155b	Serum (Bladder cancer)	[3]
Fluorescence	Wrinkled silica nanoparticles with Quantum dots	5~20 fM	37 °C	240 min	miR-96, 21, 135b	Serum (Bladder cancer)	[4]
Electrochemical	CHA and DNA walker	10 pM	37 °C	180 min	miR-155	Serum (Spike test)	[5]
Electrochemical	DNAzyme	12~25 pM	25 °C	180 min	miR-21, 141	Serum (Spike test)	[6]
SPR (Biacore X™)	CHA with streptavidin aptamer	1 pM	25 °C	90 min.	miR-21	Cell lysate	[7]
SPR	Triple-helix formation of parallel clamps	1 nM	25 °C	> 20 min	miRNA-145	NA	[8]
SPR (Biacore X™)	DNAzyme-medicated CHA and streptavidin binding	1 pM	37 °C	> 192 min	miR-21	Cell lysate	[9]
SPR imaging	Neutravidin-gold nanospheres and anti-DNA*RNA Antibody	0.5 pM	25 °C	60 min	miR-422, 223, 126, 23a	Serum	[10]
SPR (Biacore T200)	Triplex DNA nanoswitches with streptavidin-AuNPs	~ 0.8 pM	25 °C	~ 60 min	miR-183, 155	Urine (Bladder cancer)	This work

Note： CHA: Catalytic hairpin assembly

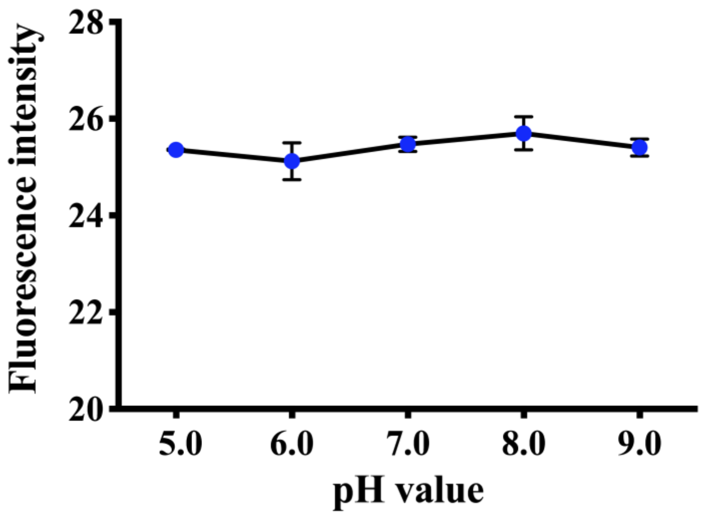

Figure S1. Performance of the pH-insensitive fluorophore (TAMRA). The experiment was conducted by measuring the fluorescence intensity of TAMRA-labeled reporter DNA (200 nM) in various pH buffers using a Varioskan LUX Multimode Microplate Reader (λ_{ex}=557 nm, λ_{em}=583 nm). The buffers consisted of 10 mM HEPES (containing 15 mM $MgCl_2$ and 80 mM NaCl), and the pH was adjusted using HCl and NaOH.

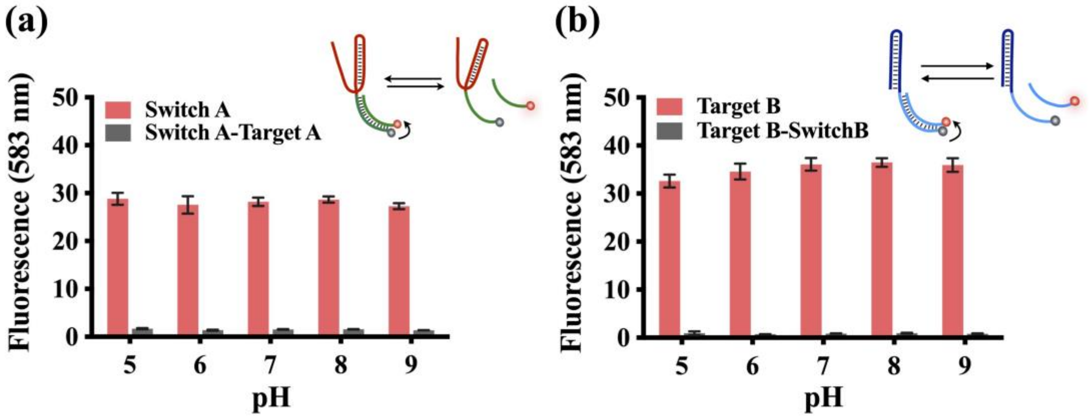

Figure S2. Evaluation of the pH stability of target/switch DNA hybrid. (a) Fluorescence intensity of target DNA-switch A from pH 5.0 to pH 9.0. (b) Fluorescence intensity of target DNA-switch B from pH 5.0 to pH 9.0. The experiments were conducted by labeling the switch DNA with a quencher (BHQ-2) and the target DNA with a fluorophore (TAMRA) at the sequence termini. After 20 min of hybridization at room temperature, the fluorescence intensities of the hybrids (200 nM) in various pH buffers were measured. The buffers consisted of 10 mM HEPES (with 15 mM $MgCl_2$ and 80 mM NaCl), and the pH was adjusted with HCl or NaOH. Error bars represent the standard deviation from N=3 experiments.

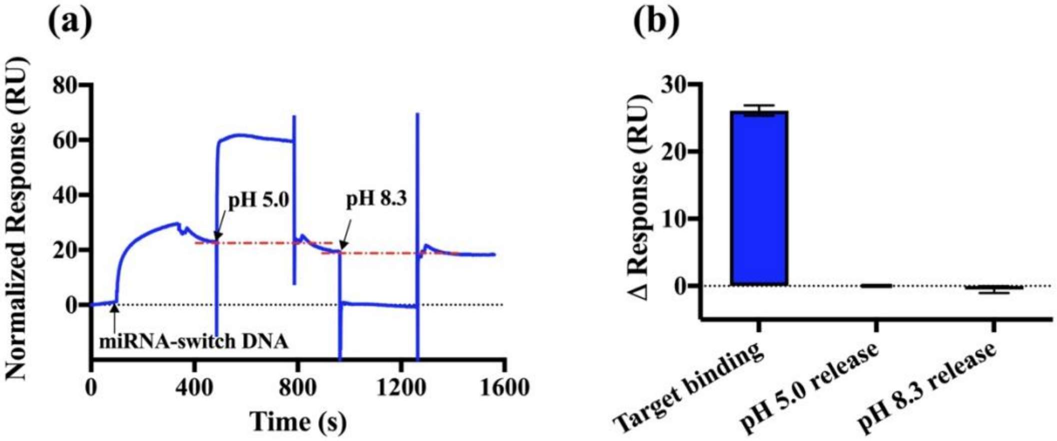

Figure S3. Binding stability of S9.6 antibody toward DNA/miRNA hybrid switches. (a) The SPR sensorgram of the switch DNA/miRNA hybrids after flowing through pH 5.0 and pH 8.3 buffers. (b) The difference in response (ΔRU) calculated before and after sample injection into the SPR system. The running buffer consisted of 10 mM HEPES (with 15 mM $MgCl_2$ and 80 mM NaCl) at pH 7.0, while the releasing buffers were 20 mM HEPES (with 5 mM $MgCl_2$ and 80 mM NaCl) at pH 5.0 and pH 8.3, respectively. After 240 s of injecting switch DNA/miRNA hybrids, pH 5.0 and pH 8.3 releasing buffers were sequentially flowed across the surface for 300 s. Error bars represent the standard deviation from N=3 experiments.

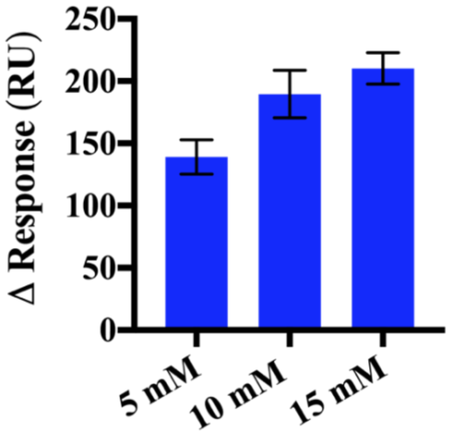

Figure S4. Optimization of $MgCl_2$ concentration in the SPR running buffer. A constant concentration of switch DNA/miRNA hybrids (50 nM) was introduced over an S9.6 antibody-immobilized chip for 240 s using a 10 mM HEPES buffer at pH 7.0, containing varying concentrations of $MgCl_2$ (5 mM, 10 mM, and 15 mM). The change in sensor response (ΔRU) was determined by comparing the signal before and after hybrid injection. Error bars represent the standard deviation from N=3 experiments.

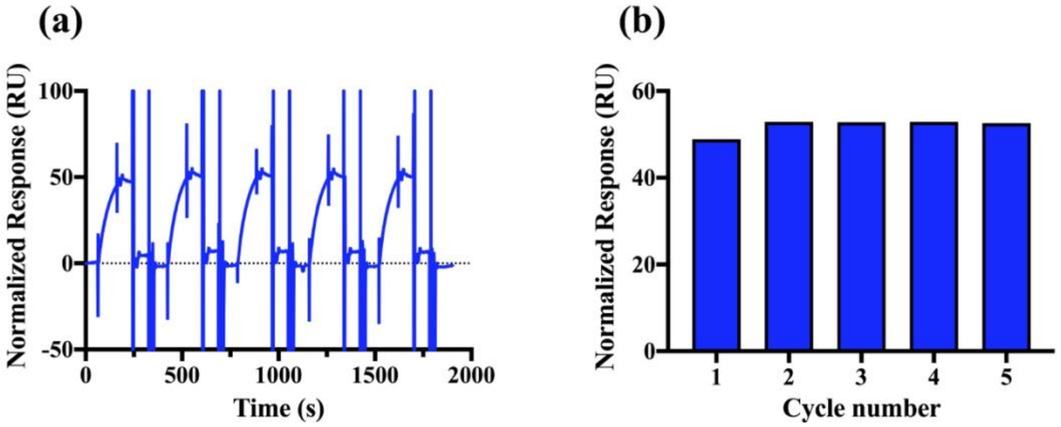

Figure S5. Regeneration efficiency of S9.6 antibody-immobilized SPR CM5 chip. (a) Switch DNA/miRNA hybrids (5 nM) were injected over the chip surface, followed by sequential regeneration using 10 mM glycine (pH 1.7) and 2 M $MgCl_2$ for 15 s each. This hybridization-regeneration process was repeated for five cycles. (b) The change in sensor response (∆RU) was measured before and after the injection of switch DNA/miRNA hybrids across the five regeneration cycles.

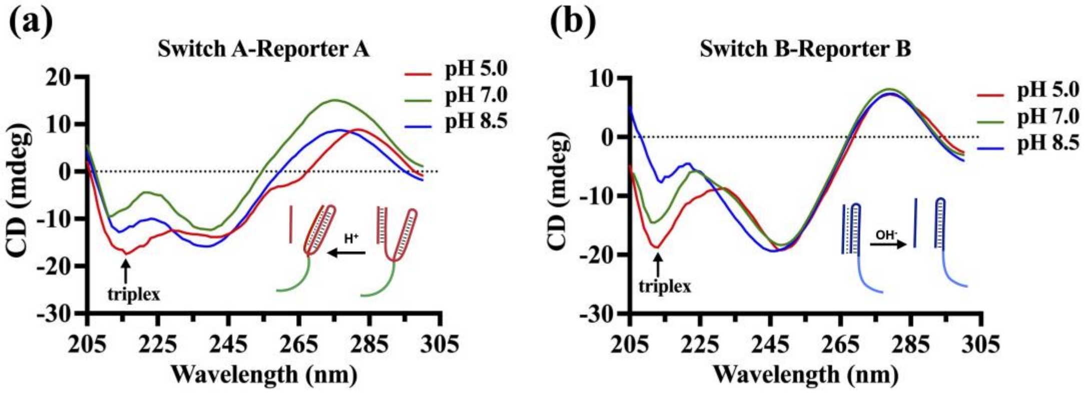

Figure S6. Circular dichroism (CD) spectroscopy method for the analysis of triplex DNA nanoswitches under different pH conditions. (a) CD spectra of triplex DNA nanoswitch A (composed of reporter A and switch A) recorded at pH 5.0, pH 7.0, and pH 8.5. (b) CD spectra of triplex DNA nanoswitch B (composed of reporter B and switch B) under identical pH conditions. For both experiments, 2 µM of reporter DNA and switch DNA were hybridized at room temperature for 30 min before measurement.

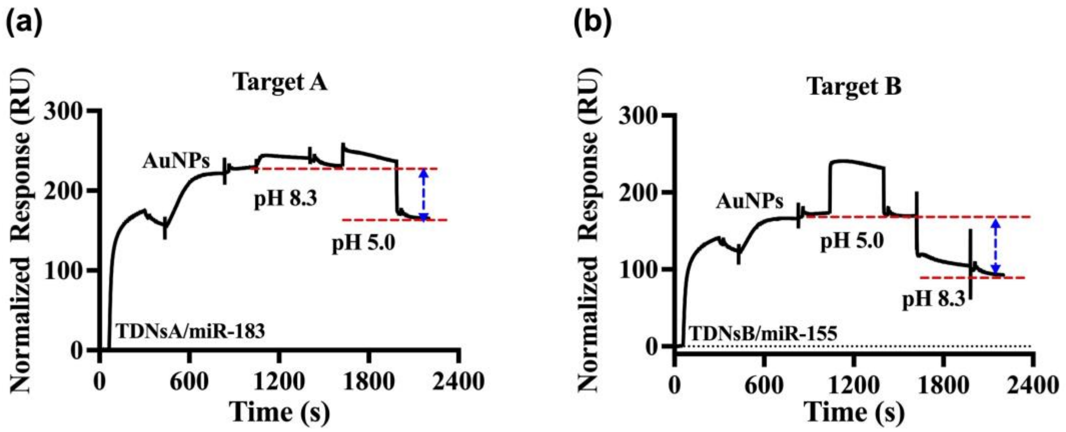

Figure S7. pH-specificity of triplex DNA nanoswitches for miRNA analysis using the SPR platform. (a) pH-specificity performance of triplex nanoswitch A functionalized with AuNPs for the detection of target A (miR-183). (b) pH-specificity performance of triplex nanoswitch B functionalized with AuNPs for the detection of target B (miR-155). In this experiment, 10 nM of miRNA and 40 nM of TDNs were used.

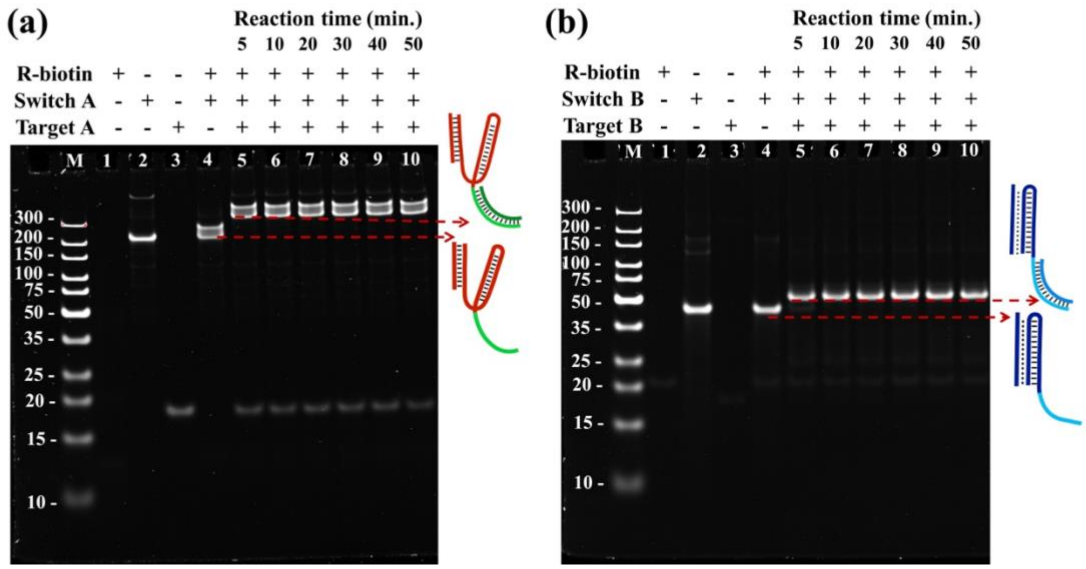

Figure S8. Optimization of target miRNAs and TDNs hybridization time using PAGE. (a) Hybridization of target A (miR-183) with TDNsA. (b) Hybridization of target B (miR-155) with TDNsB. Lane M: DNA marker (10-300 bp). Lanes 1 to 3: reporter DNA, switch DNA, and target miRNA, respectively. Lane 4: TDNs. Lanes 5 to 10: hybridization of the target miRNA with the corresponding TDNs at reaction times ranging from 5 to 50 min. All nucleic acids used in these experiments were at a concentration of 200 nM, with hybridization reactions conducted at room temperature in 10 mM HEPES buffer (15 mM $MgCl_2$, 80 mM NaCl, pH 7.0).

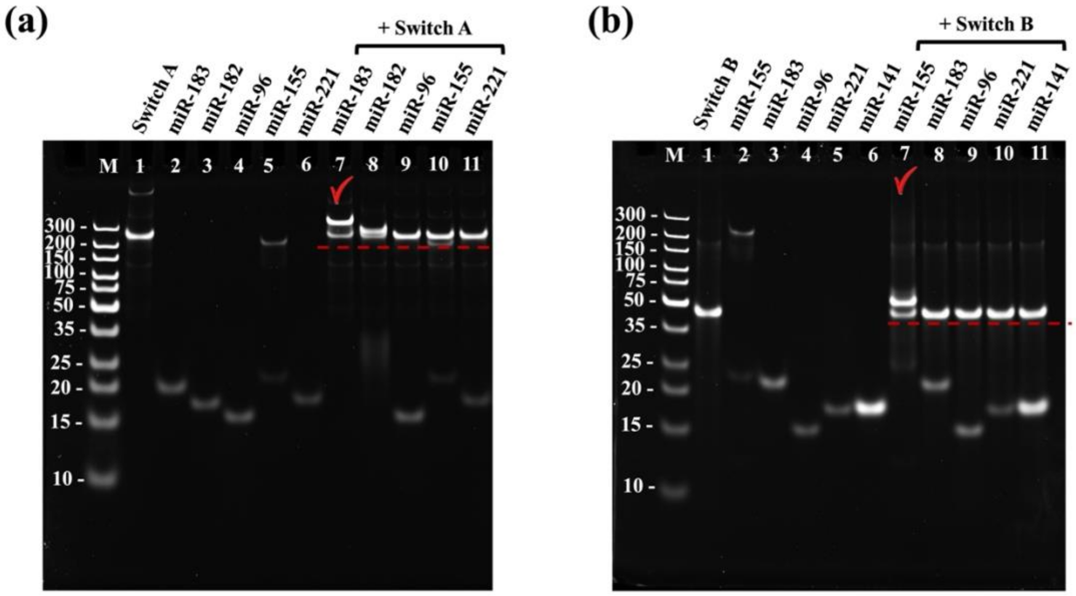

Figure S9. Specificity evaluation of TDNs via PAGE. Lane M: DNA marker (10-300 bp). Lane 1: switch DNA alone. Lane 2: specific miRNA. Lanes 3 to 6: non-specific miRNAs. Lane 7: switch DNA hybridized with the specific miRNA. Lanes 8 to 11: switch DNA hybridized with non-specific miRNAs. All nucleic acids utilized in these experiments were at a concentration of 200 nM, with hybridization reactions carried out at room temperature for 20 min in 10 mM HEPES buffer (containing 15 mM $MgCl_2$ and 80 mM NaCl, pH 7.0).

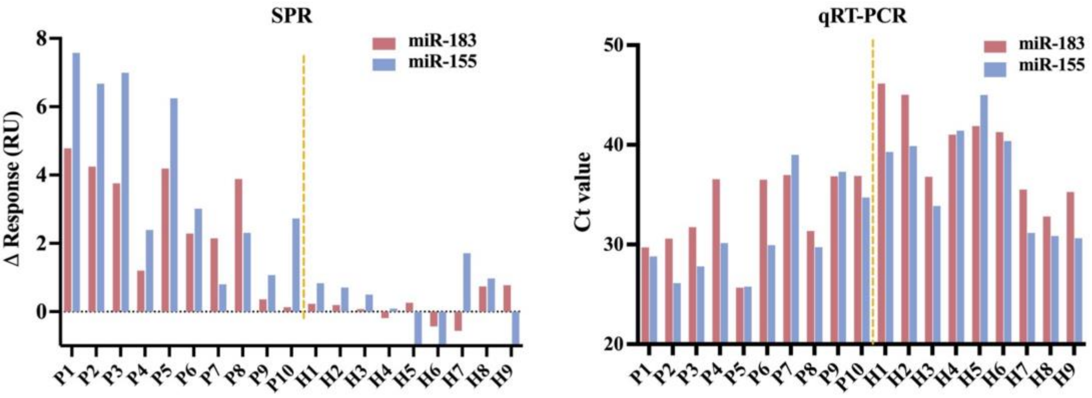

Figure S10. Analysis of microRNA levels in real urine samples determined by SPR and qRT-PCR for 10 patients and 9 healthy individuals.

REFERENCES

(1) Ma, X.; Zhou, F.; Yang, D.; Chen, Y.; Li, M.; Wang, P. miRNA Detection for Prostate Cancer Diagnosis by miRoll-Cas: miRNA Rolling Circle Transcription for CRISPR-Cas Assay. *Anal. Chem.* **2023**, *95* (35), 13220-13226. DOI: 10.1021/acs.analchem.3c02231.

(2) Xu, J.; Wei, X.; Zhang, X.; Cai, Z.; Wei, Y.; Liu, W.; Yang, J.; Li, Y.; Cai, X.; Bai, T. Multiplexed detection of bladder cancer microRNAs based on core-shell-shell magnetic quantum dot microbeads and cascade signal amplification. *Sens. Actuators B: Chem.* **2021**, *349*, 130824. DOI: 10.1016/j.snb.2021.130824.

(3) Zhang, X.; Wei, X.; Qi, J.; Shen, J.; Xu, J.; Gong, G.; Wei, Y.; Yang, J.; Zhu, Q.; Bai, T.; et al. Simultaneous Detection of Bladder Cancer Exosomal MicroRNAs Based on Inorganic Nanoflare and DNAzyme Walker. *Anal. Chem.* **2022**, *94* (11), 4787-4793. DOI: 10.1021/acs.analchem.1c05588.

(4) Wang, P.; Wei, X.; Shen, L.; Xu, K.; Wen, Z.; Gao, N.; Fan, T.; Xun, S.; Zhu, Q.; Qu, X.; et al. Amplification-Free Analysis of Bladder Cancer MicroRNAs on Wrinkled Silica Nanoparticles with DNA-Functionalized Quantum Dots. *Anal. Chem.* **2024**, *96* (12), 4860-4867. DOI: 10.1021/acs.analchem.3c05204.

(5) Meng, J.; Xu, Z.; Zheng, S.; Yang, H.; Wang, T.; Wang, H.; Zhang, Y. Development of a regenerable dual-trigger tripedal DNA walker electrochemical biosensor for sensitive detection of microRNA-155. *Anal. Chim. Acta* **2024**, *1285*, 342026. DOI: 10.1016/j.aca.2023.342026.

(6) Lin, Q.; Wu, J.; Jiang, L.; Kong, D.; Xing, C.; Lu, C. Target-driven assembly of DNAzyme probes for simultaneous electrochemical detection of multiplex microRNAs. *Analyst* **2022**, *147* (2), 262-267. DOI: 10.1039/D1AN02036F.

(7) Li, J. B.; Lei, P. H.; Ding, S. J.; Zhang, Y.; Yang, J. R.; Cheng, Q.; Yan, Y. R. An enzyme-free surface plasmon resonance biosensor for real-time detecting microRNA based on allosteric effect of mismatched catalytic hairpin assembly. *Biosens. Bioelectron.* **2016**, *77*, 435-441. DOI: 10.1016/j.bios.2015.09.069.

(8) Aviñó, A.; Huertas, C. S.; Lechuga, L. M.; Eritja, R. Sensitive and label-free detection of miRNA-145 by triplex formation. *Anal. Bioanal. Chem.* **2016**, *408* (3), 885-893. DOI: 10.1007/s00216-015-9180-6

(9) Li, X.; Cheng, W.; Li, D.; Wu, J.; Ding, X.; Cheng, Q.; Ding, S. A novel surface plasmon resonance biosensor for enzyme-free and highly sensitive detection of microRNA based on multi component nucleic acid enzyme (MNAzyme)-mediated catalyzed hairpin assembly. *Biosens. Bioelectron.* **2016**, *80*, 98-104. DOI: 10.1016/j.bios.2016.01.048.

(10) Sguassero, A.; Artiga, A.; Morasso, C.; Jimenez, R. R.; Rapun, R. M.; Mancuso, R.; Agostini, S.; Hernis, A.; Abols, A.; Line, A.; et al. A simple and universal enzyme-free approach for the detection of multiple microRNAs using a single nanostructured enhancer of surface plasmon resonance imaging. *Anal. Bioanal. Chem.* **2019**, *411* (9), 1873-1885. DOI: 10.1007/s00216-018-1331-0.
